# Polymicrobial Infections In Brain Tissue From Alzheimer’s Disease Patients

**DOI:** 10.1038/s41598-017-05903-y

**Published:** 2017-07-17

**Authors:** Diana Pisa, Ruth Alonso, Ana M. Fernández-Fernández, Alberto Rábano, Luis Carrasco

**Affiliations:** 1 Centro de Biología Molecular “Severo Ochoa” (CSIC-UAM), c/Nicolás Cabrera, 1, Universidad Autónoma de Madrid, Cantoblanco, 28049 Madrid, Spain; 2 Department of Neuropathology and Tissue Bank, Unidad de Investigación Proyecto Alzheimer, Fundación CIEN, Instituto de Salud Carlos III, Madrid, Spain

## Abstract

Several studies have advanced the idea that the etiology of Alzheimer’s disease (AD) could be microbial in origin. In the present study, we tested the possibility that polymicrobial infections exist in tissue from the entorhinal cortex/hippocampus region of patients with AD using immunohistochemistry (confocal laser scanning microscopy) and highly sensitive (nested) PCR. We found no evidence for expression of early (ICP0) or late (ICP5) proteins of herpes simplex virus type 1 (HSV-1) in brain sections. A polyclonal antibody against *Borrelia* detected structures that appeared not related to spirochetes, but rather to fungi. These structures were not found with a monoclonal antibody. Also, Borrelia DNA was undetectable by nested PCR in the ten patients analyzed. By contrast, two independent Chlamydophila antibodies revealed several structures that resembled fungal cells and hyphae, and prokaryotic cells, but most probably were unrelated to *Chlamydophila spp*. Finally, several structures that could belong to fungi or prokaryotes were detected using peptidoglycan and *Clostridium antibodies*, and PCR analysis revealed the presence of several bacteria in frozen brain tissue from AD patients. Thus, our results show that polymicrobial infections consisting of fungi and bacteria can be revealed in brain tissue from AD patients.

## Introduction

Alzheimer’s disease (AD), the leading cause of dementia in the elderly, is a neurodegenerative disease characterized by progressive dementia, neuroinflammation and neuronal death^1, 2^. An important challenge for the field is to uncover the precise etiology of AD to prevent the disease from occurring and/or to implement appropriate therapies. According to the “amyloid cascade hypothesis”, the extracellular accumulation of amyloid peptide (Aβ) triggers tau phosphorylation and cell death^3–5^. A number of laboratories have questioned the role of amyloid deposition in AD neuropathogenesis and have investigated the potential role for pathogens^6–9^. The finding that Aβ has antimicrobial activity has provided evidence to suggest that microbial infections induce the formation of Aβ-containing senile plaques^10^. Aβ peptide is known to participate in innate immune response and protects animals from fungal and bacterial infections^11^.

Herpes simplex virus type 1 (HSV-1) infection has been considered in the etiology of AD^7, 12–14^. Indeed, it was postulated more than thirty years ago that latent HSV-1 in the trigeminal ganglia could travel to different brain regions to induce AD^15^. Accordingly, HSV-1 DNA has been detected in brain samples from AD patients by PCR in different central nervous system (CNS) regions, including the frontal lobe, the temporal lobe and the hippocampus^16–19^. Interestingly, HSV-1 DNA in brains of elderly normal as well as AD patients, may not preclude a role for the virus in the disease, instead the presence of this viral DNA in the brains of apolipoprotein E4 carriers is associated with AD^20–22^. However, this correlation between HSV-1 positivity and AD has not been observed in other studies^23, 24^. Several hallmarks of the neuropathology of AD have been reproduced in culture cells. Thus, infection of neuronal cells by HSV-1 induces the synthesis and processing of β-amyloid, oxidative stress and synaptic dysfunction^25–29^. The suggestion that some bacteria, such as *Chlamydophila pneumoniae*, are involved in AD pathology has also been made^30^. Bacterial DNA has been identified by PCR in AD brain samples and bacterial morphology has been substantiated by electron microscopy and immunohistochemistry^31, 32^. Nonetheless, other research groups have been unable to demonstrate this infection in AD brains^33, 34^. The isolation of spirochetes from AD brains has been reported, leading to the suggestion that AD may in fact constitute a spirochetosis^35, 36^. Certainly, spirochetes were evidenced in AD brains using a variety of techniques, including PCR, immunohistochemistry, electron microscopy, *in situ* hybridization and culture of the bacteria^8^. Moreover, induction of β-amyloid takes place in culture neuronal cells incubated with spirochetes or lipopolysaccharide and in mice infected with *C. pneumoniae* ^37, 38^. In addition, other pathogens such as Cytomegalovirus and *Helicobacter* have been considered as the causative agent of AD^7^. Finally, the possibility that a protozoan such as *Toxoplasma gondii* is involved in AD has been posited^39^, but not experimentally supported^40, 41^.

Our group has provided extensive evidence for fungal infection in patients with AD^42–46^. Proteomic analyses identified proteins from a range of fungi in AD brain and DNA amplification rendered a number of fungal species present in this tissue^43^. Furthermore, visualization of fungal cells and hyphae using immunohistochemistry with specific antifungal antibodies clearly point to fungal infection in different regions of the CNS^45, 46^. In the present work, we tested whether other pathogens could be detected together with fungal structures using similar techniques, i.e. immunohistochemistry and PCR with the same AD samples.

## Results

### Analysis of the specificity of the different antibodies

We tested several commercial antibodies against HSV-1 and a range of bacteria. In addition, we used in-house-generated polyclonal rabbit antibodies against *C. albicans* and rat polyclonal antibodies against *T. viride* ^45, 47^. We first assayed their specificity and sensitivity using immunohistochemistry. Two mouse monoclonal antibodies, which recognized HSV-1 ICP0 (early viral protein) and ICP5 (late viral protein), were tested in HeLa cells infected with HSV-1 (5 pfu/cell). A second rabbit polyclonal antibody against the human initiation factor of translation eIF4GI was also used. Mock- or HSV-1-infected HeLa cells were processed at different times after infection. Supplementary Figure 1 shows that the monoclonal antibody against ICP0 (green) specifically immunolabeled infected cells, whereas eIF4GI imunoreactivity was found in the cytoplasm (red) and DAPI stained the nuclei (blue). ICP0 immunoreactivity was found inside the nucleus at 3 hours post infection (hpi). However, at later times (6 hpi) immunoreactivity was also located in the cytoplasm, in good agreement with the observations reported for this viral protein^44^. Immunostaining for ICP5 was positive at later times of infection (18 hpi), but not at earlier times (3 hpi). No HSV-1 immunostaining was observed in mock-infected cells.

Two *Borrelia* antibodies were tested against a commercial Euroimmun kit containing *B. burgdorferi*. One was a rabbit polyclonal antibody that, according to the manufacture, recognizes several proteins by western blotting, whereas the other was a mouse monoclonal antibody selective for a *Borrelia* protein of 28 kDa. Both antibodies immunoreacted with *Borrelia*, whereas antibodies to *Chlamydophila* and *C. albicans* did not recognize *B. burgdorferi*. Some immunostaining was also apparent with the *T. viride* antibody (Supplementary Figure 2, panel A).

Two antibodies were tested against a Euroimmun kit containing *C. pneumonie*. One was a rabbit polyclonal antibody against the major outer membrane porin protein of *C. pneumoniae* and the other was a mouse monoclonal antibody against *C. trachomatis* that recognized lipopolysaccharide. Only the rabbit polyclonal antibody recognized *C. pneumoniae*, whereas the mouse monoclonal antibody did not immunoreact with these samples (Supplementary Figure 2, panel B). Curiously, the rabbit polyclonal antibody against *Borrelia* also immunoreacted with *C. pneumoniae* and some immunostaining was apparent with the *C. albicans* antibody.

To further test the specificity of the in-house antibodies, we evaluated their reactivity against a Euroimmun kit containing five species of *Candida*: *C. albicans*, *C. parapsilosis*, *C. glabrata*, *C. tropicalis,* and *C. krusei*. Supplementary Figure 3 shows that the *C. albicans* antibody recognized all five *Candida* species. The rat polyclonal antibody against *T. viride* recognized, with less intensity, *C. albicans*, *C. glabrata* and *C. tropicalis*, but not *C. parapsilosis* or *C. krusei.* Moreover, the *Borrelia* polyclonal antibodies immunoreacted robustly with *C. glabrata* and *C. tropicalis* and also to some extent with *C. krusei* and *C. parapsilosis*, but not with *C. albicans* (Supplementary Figure 4). Furthermore, the rabbit polyclonal antibody against *C. pneumoniae* immunoreacted with all five *Candida* species tested, whereas the mouse monoclonal antibodies specific for *Borrelia* or *C. pneumoniae* did not react with any of the *Candida* species analyzed.

Two additional antibodies against bacteria were tested. One was a rabbit polyclonal antibody against *C. perfringens* that, according to the manufacturer, can also immunoreact with other bacteria. The other was a mouse monoclonal against peptidoglycan, the major component of the cell wall of Gram-positive bacteria. The immunoreactivity of these two antibodies against *Borrelia*, *C. pneumoniae* and several *Candida spp* is shown in Supplementary Figure 5. Notably, the *C. perfringens* antibody immunoreacted with *Borrelia* and also with *C. parapsilosis*, *C. tropicalis* and *C. krusei*, while the anti-peptidoglycan antibody recognized *C. pneumoniae*, *C. parapsilosis* and *C. tropicalis* (very poorly). In conclusion, some commercial anti-bacteria antibodies can recognize not only prokaryotic cells, but also some fungal species. It is therefore important to determine their specificity.

### Testing HSV-1 proteins and DNA in brain tissue

**Figure 1:**
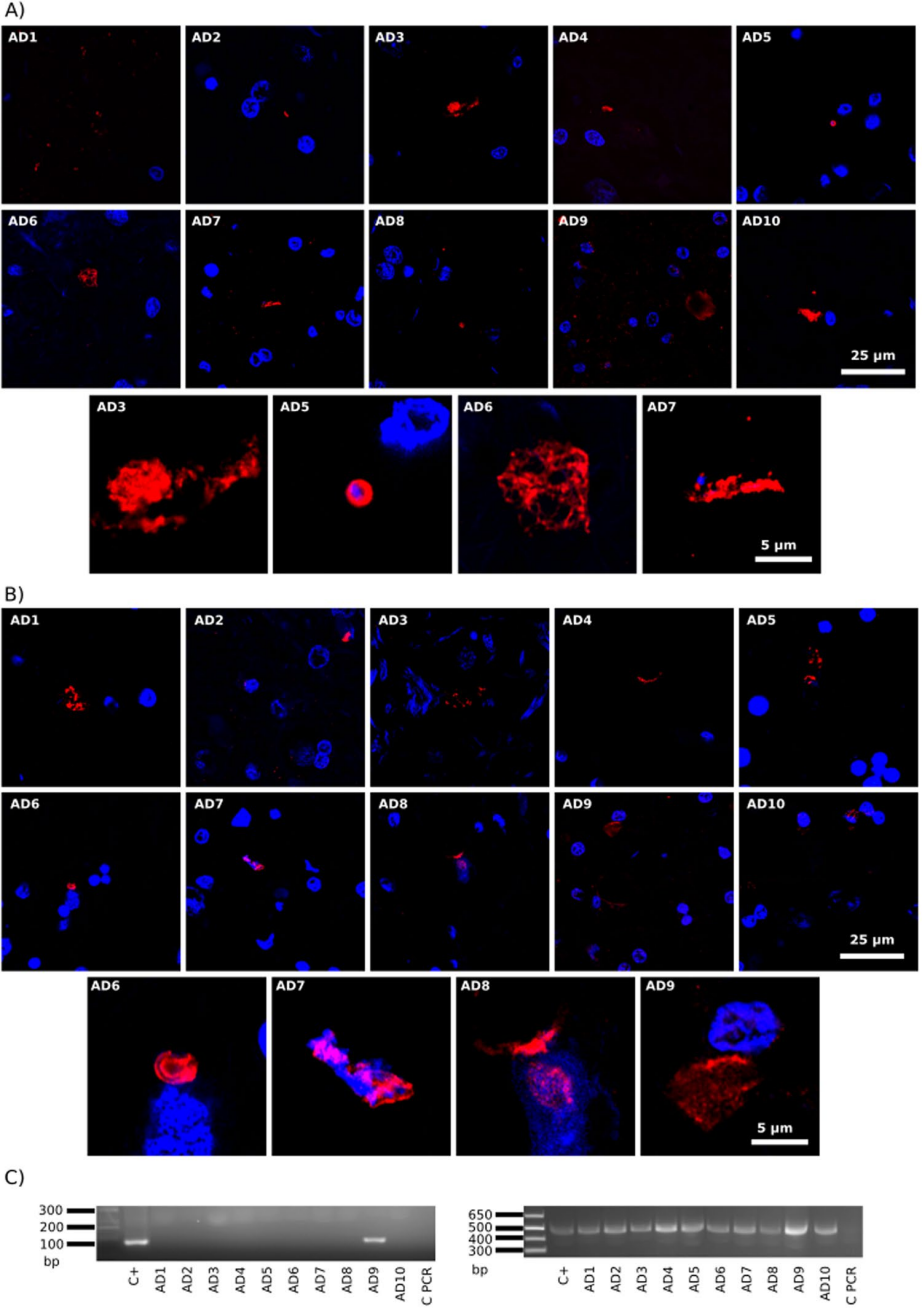
Immunohistochemistry of HSV-1 proteins in brain sections from ten AD patients and PCR analysis of HSV-1 DNA. Entorhinal cortex/hipoccampus (ERH) sections from ten AD patients. Panel A: samples were immunostained with mouse monoclonal antibody (1:50) against HSV ICP0 (green), and rabbit polyclonal antibody (1:100) against *C. albicans* (red). The different patients are numbered from AD1 to AD10 and one field is shown for each patient. In addition, four selected sections are shown at higher magnification below the ten AD patients. Panel B: samples were immunostained with mouse monoclonal antibody (1:50) against HSV-1 ICP5 (green) and rabbit polyclonal antibody (1:100) against *C. albicans* (red). DAPI staining of nuclei appears in blue. Scale bar as shown in the figure. Panel C: PCR analysis of HSV-1 and β-globin DNA in frozen brain tissue from ten AD patients. Left panel: Nested PCR analysis of ten ERH samples using primers that amplify HSV-1 *glycoprotein D* gene. The primers employed were HSV-1 FE (forward external) and HSV-1 RE (reverse external) for the first PCR and primers HSV-1 FI (forward internal) and HSV-1 RI (reverse internal) for the second PCR. As positive control, DNA from HSV-1-infected HeLa cells was used. Right panel: PCR analysis using β-globin oligonucleotide primers. As positive control, DNA extracted from HeLa cells was used. Control PCR: PCR without DNA. DNA markers are indicated on the left.

Our initial goal was to examine the entorhinal cortex/hippocampus (ERH) region from ten patients with AD to test the possibility that HSV-1 reactivation occurred. Tissue sections of ERH were immunostained with antibodies against ICP0 or ICP5, and also an antibody against *C. albicans*. Immunohistochemistry of brain sections failed to detect the HSV-1 early (ICP0) or late (ICP5) proteins (Fig. 1A,B; green), whereas the *C. albicans* antibody immunostained the typical yeast-like and hyphal morphologies previously described by us (Fig. 1A,B; red). These findings suggest that herpetic reactivation did not occur in the brain of the AD patients tested. Of note, it is well established that the ERH region is usually the first and more affected brain region in AD^48^.

A complementary approach to assess the existence of HSV-1 in AD brains is by nested PCR of DNA extracted from frozen ERH tissue. Thus, DNA was extracted from frozen samples of the ten AD patients and nested PCR was performed on the HSV gene glycoprotein D^18^. As a control, DNA extracted from infected HeLa cells was also used as a template. Supplementary Figure 6 shows the HSV-1 DNA fragments amplified by each set of primers, by individual or by nested PCR. Also shown are the amplified bacterial DNA fragments generated from the corresponding primers (see below). Results showed that only one of the ten patients examined was positive for an amplified HSV-1 DNA fragment (Fig. 1C left panel, patient AD9). As a control for DNA in the samples, we used primers to amplify the human β-globin, which was present in all samples (Fig. 1C, right panel). Sequencing of the glycoprotein D fragment confirmed that it belonged to HSV-1 (98% identity). This finding is consistent with results indicating that herpes DNA is present in a low proportion of brain samples^23, 24^ As we examined only a small region of the brain, it is possible that the frequency of HSV-1 DNA occurrence would increase if more regions of CNS were examined. In previous works, HSV-1 DNA has been examined in different CNS regions including the frontal and temporal lobes and the size of patients examined was higher^12, 13, 49^. However, the appearance of HSV-1 DNA does not indicate viral reactivation because this technique does not distinguish between viral latency and productive infection.

### Testing *Borrelia* macromolecules in brain tissue

To assess the possibility that spirochaetosis can be observed in AD brains, the two antibodies against *B. burgdorferi* were tested in brain tissue. ERH sections from the ten AD patients were incubated with rabbit polyclonal or mouse monoclonal *B. burgdorferi* antibodies. A second (antifungal) antibody was also employed; in the case of the *Borrelia* polyclonal antibody, the antifungal antibody was a rat polyclonal antibody against *T. viride*, whereas the mouse monoclonal antibody was tested in combination with a rabbit polyclonal antibody against *C. albicans*.

**Figure 2:**
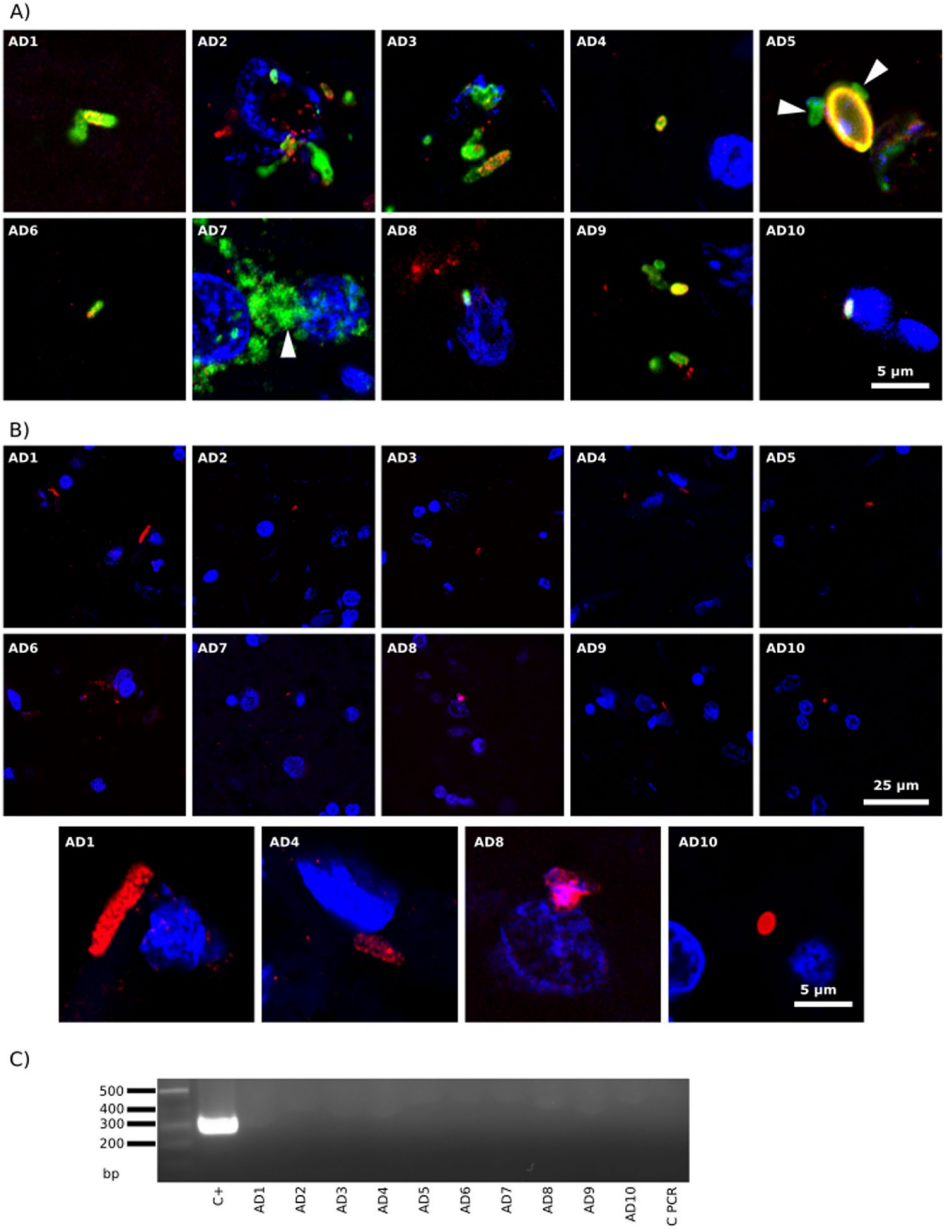
Detection of *Borrelia* proteins and DNA in ERH samples from ten AD patients. PCR assay of *B. burgdorferi* DNA. Panel A: ERH sections were immunostained with rabbit polyclonal antibody (1:50) against *B. burgdorferi* (green) and rat polyclonal antibody (1:20) against *T. viride* (red). Panel B: ERH sections were immunostained with mouse monoclonal antibody (1:10) against *B. burgdorferi* (green) and rabbit polyclonal antibody (1:100 dilution) against *C. albicans* (red). DAPI staining of nuclei appears in blue. Scale bar as shown in the figure. Panel C: PCR analysis of *Borrelia* DNA in frozen brain tissue from ten AD patients. Nested PCR analysis of ten ERH samples using Borr primers to amplify *flagellin* gene. The primers employed were Borr FE–Borr RE for the first PCR and primers Borr FI–Borr RI for the second PCR. As positive control, DNA extracted from *B. burgdorferi* was used. Control PCR: PCR without DNA. DNA markers are indicated on the left.

While the two *Borrelia* antibodies immunoreacted robustly with *B. burgdorferi* in our initial specificity testing (Supplementary Figure 2, panel A), the characteristic morphology of spirochetes was not found after extensive analysis of ERH brain sections (Fig. 2A, green). However, the *Borrelia* polyclonal antibody immunoreacted with a variety of structures, some of which were yeast-like and contained nuclei (DAPI-positive). Some of these structures also immunoreacted with the *T. viride* antifungal antibody (red), whereas other smaller structures did not immunoreact and, in principle, could be prokaryotic in origin (see arrows in Fig. 2A, panels AD5 and AD7). It seems clear that the *Borrelia* polyclonal antibody recognizes fungal, and perhaps prokaryotic structures that are not related morphologically to spirochetes. These structures were not detected when the monoclonal *B. burgdorferi* was used (Fig. 2B). In fact, the monoclonal did not immunoreact with any cell in the brain sections, despite the fact that it recognized *B. burgdorferi* in our initial specificity testing (Supplementary Figure 2). By contrast, the *C. albicans* rabbit polyclonal antibody immunoreacted with a variety of rounded and hyphal structures (Fig. 2B, red), as we have recently described^45–47^.

To further assess the possibility of spirochetosis, we performed nested PCR on extracted DNA using primers for *Borrelia spp* flagellin gene. As expected, the corresponding DNA fragments were detected after amplification of a control sample, however, no product was obtained from the DNA extracts from the ten patients (Fig. 2C), further supporting the view that spirochetosis is not evident in these patients.

### Testing *Chlamydophila* macromolecules in brain tissue

**Figure 3:**
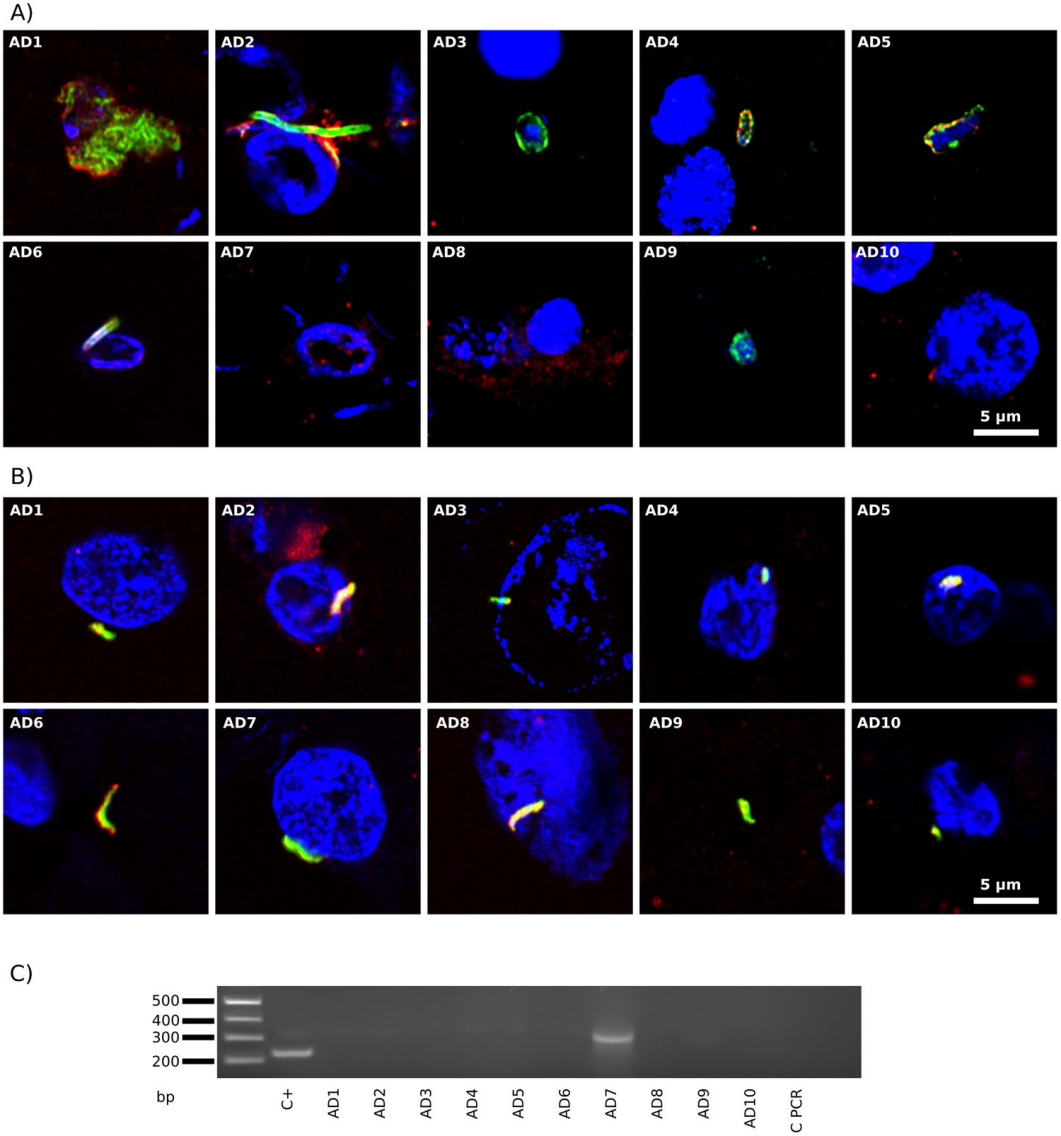
Immunohistochemistry and PCR analysis to detect *C. pneumoniae* proteins and DNA in ERH samples from AD patients. Panel A: ERH sections were immunostained with rabbit polyclonal antibody (1:20) against *C. pneumoniae* (green) and rat polyclonal antibody (1:20) against *T. viride* (red). Panel B: samples were immunostained with mouse monoclonal antibody (1:10) against *C. pneumoniae* (green) and rabbit polyclonal antibody (1:100) against *C. albicans* (red). DAPI staining of nuclei appears in blue. Scale bar as shown in the figure. Panel C: PCR analysis of *C. pneumoniae* DNA in frozen brain tissue from ten AD patients. PCR analysis of ten ERH samples using primers Clam to amplify MOMP gene. The primers employed were Clam FE–Clam RE for the first PCR and primers Clam FI–Clam RI for the second PCR assay. As positive control, DNA extracted from *C. pneumoniae* was used. Control PCR: PCR without DNA. DNA markers are indicated on the left.

We have also explored the potential infection of AD brain by *C. pneumoniae* using immunohistochemistry and PCR. Two commercial *C. pneumoniae* antibodies were tested. The polyclonal antibody cross-reacted with five yeast species in preliminary tests (Supplementary Figure 4). Consistent with this, we found yeast-like and hyphal structures in ERH sections from several AD brains (Fig. 3A, green). Some of these structures were also labeled with the *T. viride* antibody (red), suggesting a fungal origin. A different picture emerged using the mouse monoclonal antibody against lipopolysaccharide. In this case, several prokaryotic-like forms were detected intranuclearly or close to the nucleus (green) (Fig. 3B, all panels except AD6 and AD9), suggesting that they represent intracellular bodies, although they do not resemble the elementary or reticulate bodies of *C. pneumoniae*. Nevertheless, staining with an antibody against *C. albicans* revealed that these structures were also immunopositive (red), at least in part. Thus, the two *C. pneumoniae* antibodies failed to categorically detect the presence of *C. pneumoniae*, but a variety of different forms were immunopositive, some of which clearly represented fungal structures and others possibly prokaryotic cells.

As before, we performed nested PCR on extracted DNA using specific primers that amplified sequences in the MOMP gene of *C.* *pneumoniae*. A DNA fragment slightly higher in size to the predicted amplification product was detected in only one out of ten patients (Fig. 3C, patient AD7). Sequentiation of this fragment revealed that it was of human origin.

### Searching for other bacteria in AD brains

**Figure 4:**
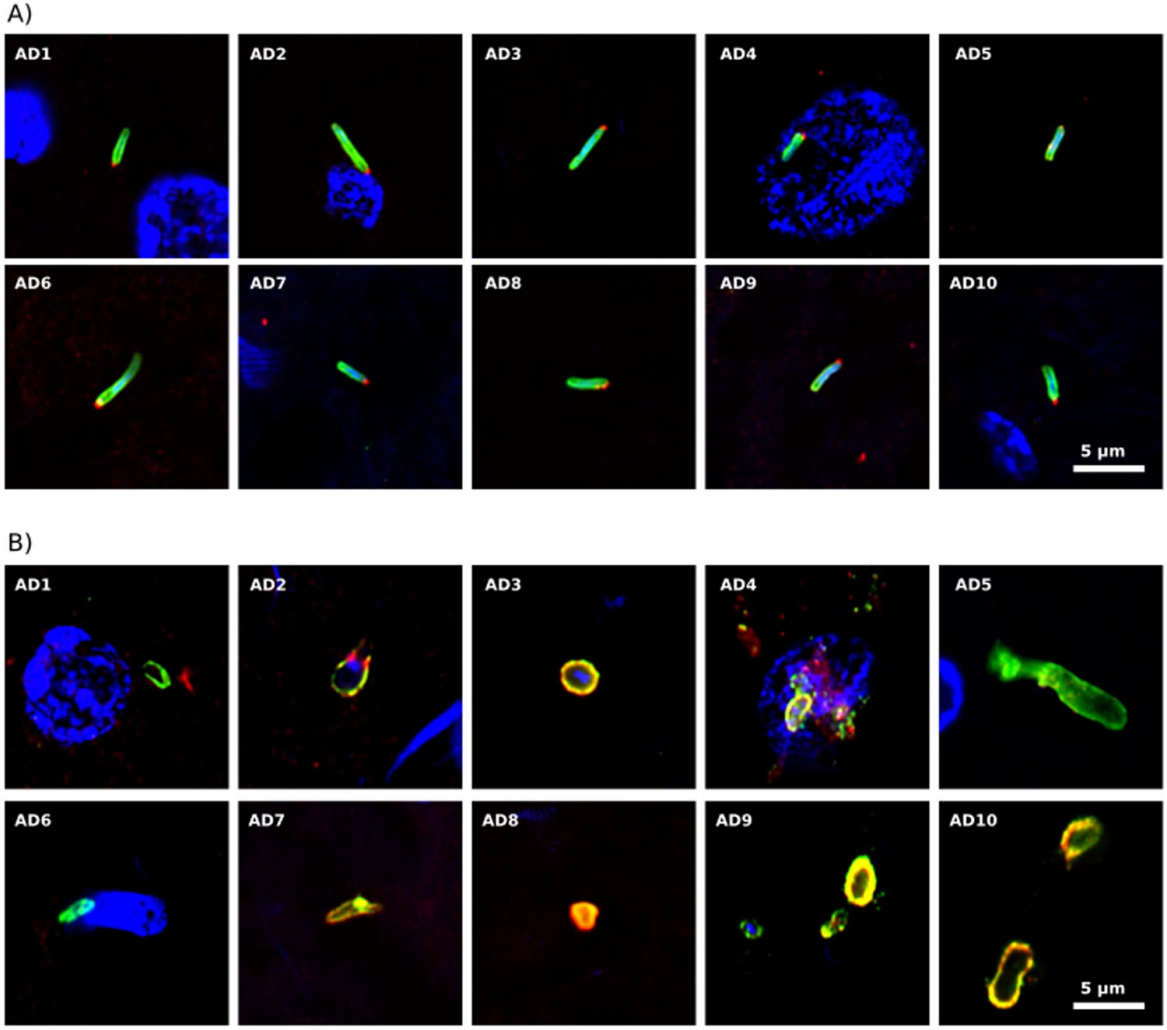
Immunohistochemistry of bacterial and fungal proteins in ERH sections from AD patients. Panel A: samples were immunostained with rabbit polyclonal antibody (1:20) against *C. pneumoniae* (green) and rat polyclonal antibody (1:20) against *T. viride* (red). Panel B: samples were immunostained with rabbit polyclonal antibody (1:20) against *C. perfringens* (green) and rat polyclonal antibody (1:20) against *T. viride* (red). DAPI staining of nuclei appears in blue. Scale bar as shown in the figure.

We observed that double staining ERH sections with a rabbit polyclonal *C. pneumoniae* antibody and a rat polyclonal *T. viride* antibody revealed a prokaryotic-like structure of 3–5 μm (Fig. 4A). These cells were clearly stained with the *C. pneumoniae* antibody (green), and curiously the *T. viride* antibody labeled a red dot on one “pole” of the cell, perhaps due to polar flagella. This staining pattern and morphology was found in ERH sections from all ten AD patients examined (Fig. 4A). We attempted to confirm this staining with a commercial rabbit polyclonal antibody against *Clostridium*, which can also immunoreact with other bacteria. This antibody was also combined with the rat polyclonal antibody against *T. viride*. Once again, we found a variety of structures in each ERH section from the ten AD patients examined (Fig. 4B). Some of these structures could be fungal since the *Clostridium* antibody also immunoreacts with yeast cells (Supplementary Figure 5). However, we did not observe the morphology seen in Fig. 4A. In one ERH sample, a number of cytoplasmic green or red dots were observed, but each dot was only labeled with one antibody (see Fig. 4B, panel AD4). In other instances, hyphal structures (panel AD5) or yeast-like cells positive for DAPI (panels AD2, AD3 and AD9) were also seen.

**Figure 5:**
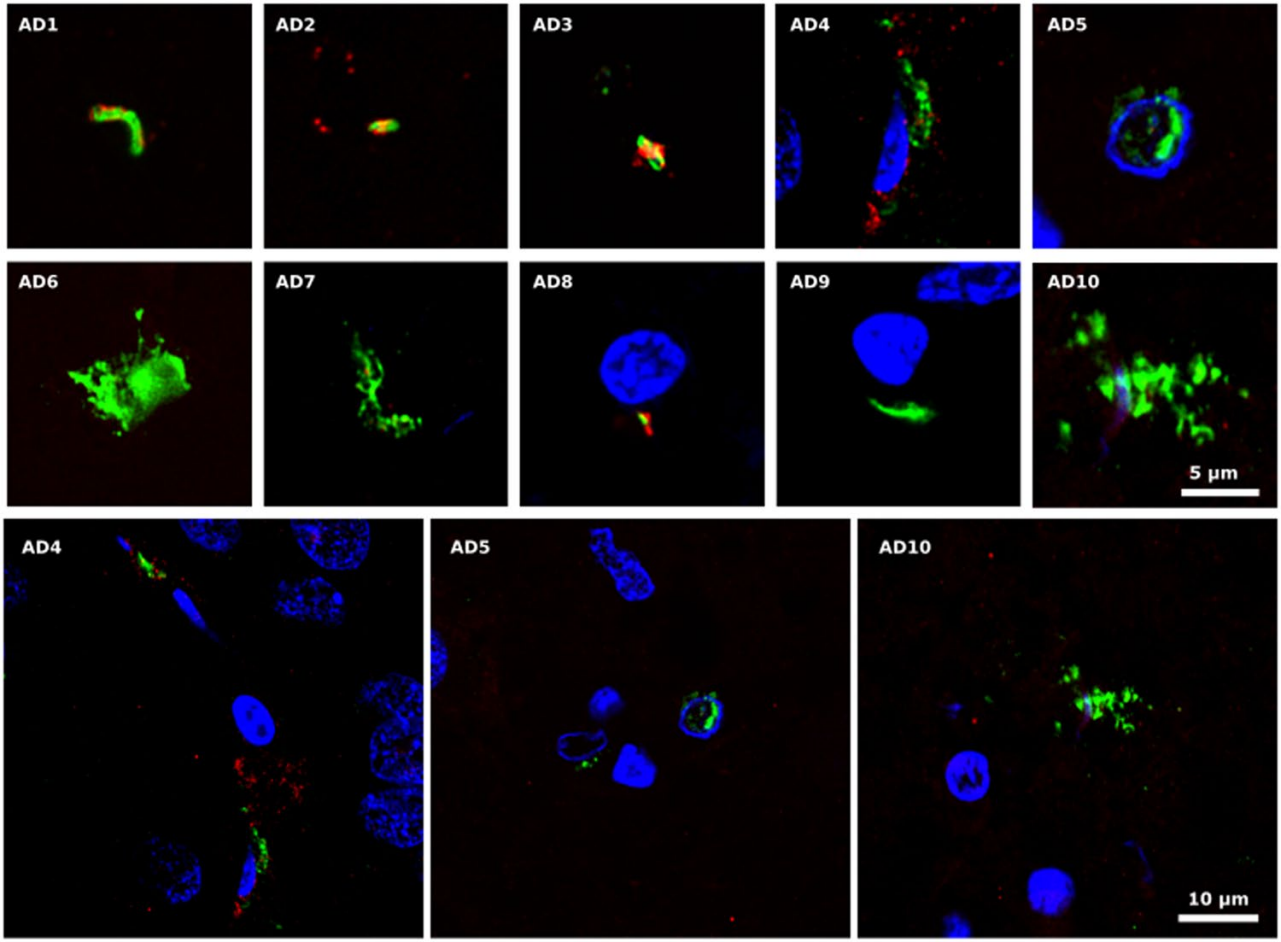
Peptidoglycan in ERH sections from AD patients. ERH samples were processed as described in Materials and Methods. Samples were first immunostained with mouse monoclonal antibody (1:20 dilution) against peptidoglycan (green) and afterwards with rabbit polyclonal antibody (1:50 dilution) against *C. albicans* (red). DAPI staining of nuclei appears in blue. Scale bar as shown in the figure.

We further explored the possibility of bacterial infection in ERH sections using double staining with a mouse monoclonal antibody against peptidoglycan (green) and the *C. albicans* rabbit polyclonal antibody (red). Several structures were also detected with this combination, but it was unclear if they belonged to fungal or prokaryotic cells (Fig. 5). Curiously, a number of dots appeared close to the nucleus in patient AD4, while two cells, perhaps bacteria, were observed inside a nucleus in patient AD5. In summary, the use of different antibodies reveals a variety of structures that, in some cases, could be prokaryotic in origin, but in the majority of occasions they resemble fungi.

### PCR assays to detect microbial DNA in brain tissue from AD

**Figure 6:**
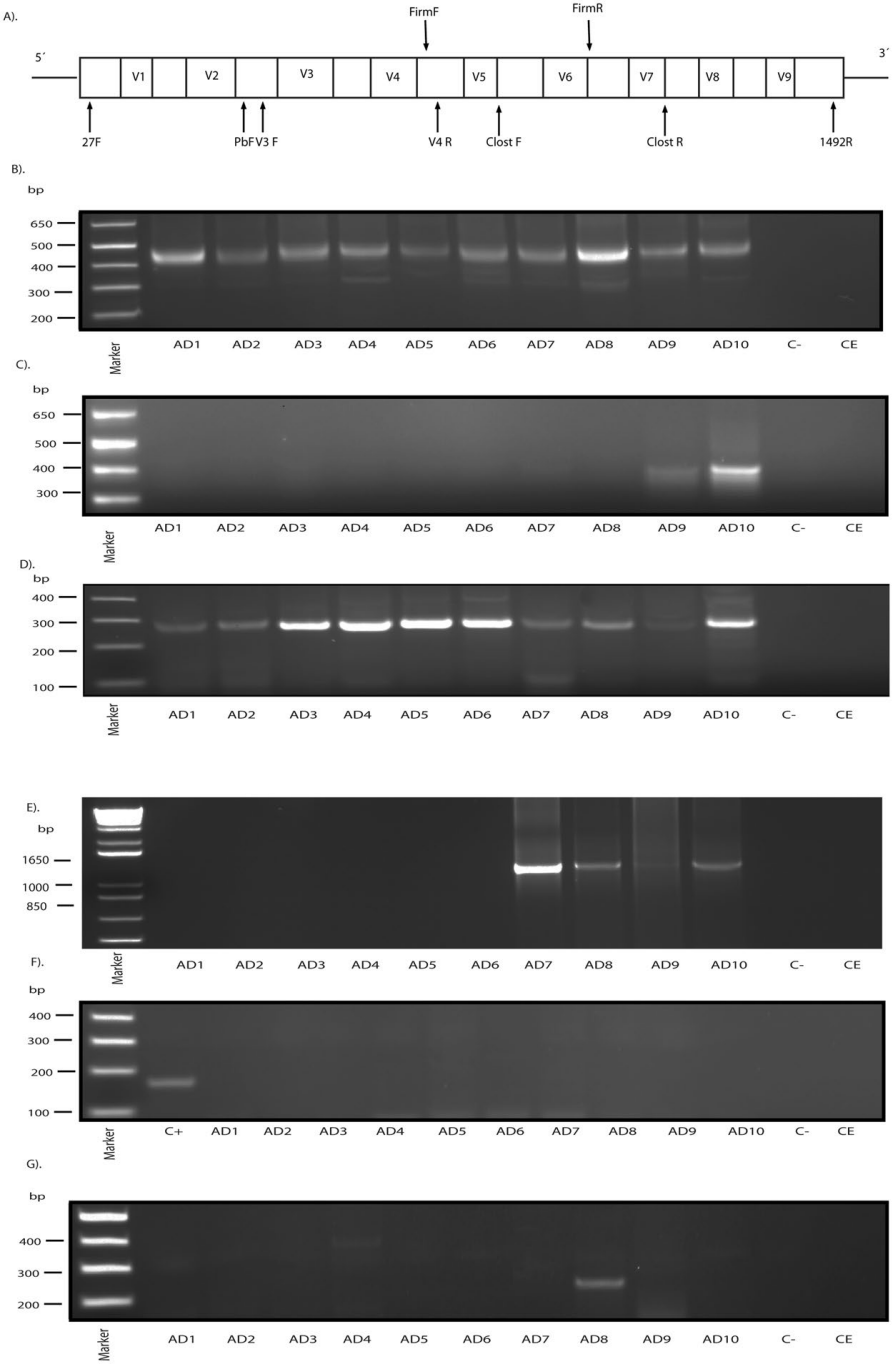
PCR analysis of different microorganisms in brain tissue from ten AD patients. Panel A: Schematic representation of bacterial 16 S rRNA gene with the different variable regions (V1–V9). Location of the primers employed for the different nested PCR carried out in this study: 27 F and 1492 R for the first PCR and the second PCR with the different sets of primers: V3F-V4R (universal primers), FirmF-FirmR (*Firmicute*s primers), Clost F-Clost R (*Clostridium* primers), P(b) F-1492R (*Bacillus* primers). F: forward; R: reverse. Panels B–E: Agarose gel electrophoresis of the DNA fragments amplified by nested PCR of DNA obtained from frozen ERH tissue from ten AD patients. Panel B: Amplification of bacterial DNA fragment using universal oligonucleotide primers by nested PCR. The primers employed were 27F-1492R for the first round PCR and V3-V4 for the second PCR. All samples generated a product of 400 bp. Panel C: Amplification of *Firmicutes* DNA fragments using specific primers by nested PCR. The primers employed were 27F-1492R for the first PCR and Firm F-Firm R for the second PCR. AD9 and AD10 show a product of about 400 bp. Panel D: Identification of *Clostridium* spp. DNA by direct PCR. The primers employed were Clost F-Clost R. All the patients show a product of 300 bp. Panel E: Nested PCR assay to amplify Bacillus spp. The primers employed were 27F-1492R for the first PCR and P(**b**) F-1492 R for the second PCR. AD7, AD8 and AD10 show a product of about 1100 bp. Panel F: Direct PCR analysis to amplify the *gadB* locus of *E.coli*. The primers employed were *E.coli* F and *E.coli* R. As positive control (C+), DNA was extracted from *E.coli*. Panel G: Nested PCR analysis to amplify *SAG2* partial gene of T. gondii. The primers used were Toxop FE and Toxop RE in the first PCR and Toxop FI and Toxop RI for the second PCR. AD8 amplified a product of about 260 bp. Control -: PCR without DNA. CE: Control of DNA extraction without brain DNA. DNA markers are indicated on the left.

We next sought to identify the potential bacteria in ERH samples from AD patients by PCR. To this end, a battery of primers were designed and tested against DNA from the ten AD patients (see Supplementary Table II). Initially, we used nested PCR with universal primers that amplify a sequence internal to the16S rRNA gene (see scheme Fig. 6A). Several DNA fragments were amplified (Fig. 6B). Sequencing analysis revealed that some of them were human in origin, whereas a 400 bp fragment, which was amplified in all ten patients, corresponded to different bacteria that depended on the patient (Supplementary Table III). For clarity we only report the bacterial species with the highest identity. The bacterial species found corresponded to the following: *Burkholderia spp* (AD1, AD5, AD8, AD9 and AD10) with an identity of 89–98%, uncultured *Sphingomonas* (AD2) with an identity of 85%, *Brevibacillus spp* (AD3) with an identity of 85%, and *Xanthomonadaceae bacterium* (AD6 and AD7) with an identity of 90% and 87%, respectively. It should be noted that the *Burkholderia* genus has been derived from the *Pseudomonas genus*. Also, nested PCR was performed with primers designed to amplify species of the *Phylum Firmicutes*. One DNA fragment of about 300 bp was amplified from DNA of AD9 and AD10 (Fig. 6C). After sequencing, the fragments corresponded to *Staphylococcus epidermidis* (94%) and *Stenotrophomonas maltophilia* (99%), respectively. A direct PCR assay was also carried out using primers to amplify a DNA region inside the *Clostridium* 16 S rRNA gene. Notably, a clear DNA fragment of about 300 bp was amplified in all ten AD patients (Fig. 6D). After sequencing, this DNA fragment corresponded to *Burkholderia spp* in all patients except AD1, AD7 and AD9. An additional nested PCR was performed using primers designed to amplify *Bacillus spp*, generating a DNA fragment of about 1100 bp in AD7, AD8 and AD10 (Fig. 6E). Sequencing of this fragment identified uncultured *Streptococcus* in AD7 and AD8 with an identity of 97 and 92%, respectively, and *Staphyloccocus* *epidermidis* in AD10 with an identity of 94%. Recently, it has been reported that *E. coli* can be detected in brain tissue from AD patients^50^. To test this possibility, we performed PCR using the primers described by these authors to amplify a region in the gadB gene of *E. coli*. Figure 6F shows that whereas the PCR was positive with a control sample of *E. coli* DNA, no fragment was amplified in the ten AD DNA samples. Finally, the possibility that the protozoan *T. gondii* could be involved in the etiology of AD has been considered^39^. However, seroprevalence of antibodies against this protozoan is similar in controls and AD patients^40, 41^. Therefore, we used nested PCR using primers to amplify the SAG2 locus^50^. A DNA fragment of about 280 bp was found only in AD8 (Fig. 6G), and sequencing revealed it to be human.

### Analysis of HSV-1 and bacteria in brain samples from control subjects

We have carried out a parallel analysis on ERH sections using immunohistochemistry (eight control subjects) and PCR (seven control subjects). We first tested for the presence of HSV-1 ICP0 and ICP5 protein and *C. albicans* as described in Fig. 1. No HSV-1 proteins were found in the control subjects (Supplementary Figure 7A,B). Also, yeast-like cells immunolabeled with the *C. albicans* antibody were observed only very occasionally (Supplementary Figure 7A,B), indicating their rarity in control subjects. These findings are in good agreement with the results from our previous study comparing the burden of fungal infection in AD patients and controls^47, 51^. HSV-1 DNA (glycoprotein D) gene was positive in two out of seven controls (Supplementary Figure 7C).

Immunohistochemistry with *Borrelia* antibodies revealed a few yeast-like structures (green) in some ERH sections from controls with the rabbit polyclonal antibody (Supplementary Figure 8A). Some of these structures were also immunolabeled with the *T. viride* antibody (red), suggesting that they could represent fungal cells. In agreement with the results described in Fig. 2, no antigens were detected with the mouse monoclonal antibody against *Borrelia* (Supplementary Figure 8B). However, a few fungal-related structures were detected with the *C. albicans* antibody (red) in some controls, whereas others were totally devoid of immunoreactivity (Supplementary Figure 8B). Finally, spirochaetal DNA was not found in any of the seven controls (Supplementary Figure 8C).

Several interesting findings were noted when using two *C. pneumoniae* antibodies. First, the rabbit polyclonal antibody stained a few yeast-like and hyphal structures (green) that in some instances were also immunostained with the *T. viride* antibody (red) (Supplementary Figure 9A). These fungal structures were rare and in some of them, such as in C1, C4, C5 and C8, the nucleus was evident by DAPI staining. A similar finding was observed when the mouse monoclonal antibody was tested in combination with the rabbit polyclonal *C. albicans* antibody (Supplementary Figure 9B). Rounded yeast cells and hyphae could be detected with the *C.* *pneumoniae* antibody, although they were much less abundant than those observed in AD patients (Fig. 3). The presence of *C.* *pneumoniae* DNA was analyzed by nested PCR and only in one case (C1) could a faint band be seen (Supplementary Figure 9C), which corresponded to *C. pneumoniae* by sequencing.

*Clostridium* and peptidoglycan antibodies were tested on control brains following a protocol similar to that described in Figs 4B and 5. Very few structures were immunolabeled with these antibodies in five controls (green), whereas no immunolabeling appeared in the remaining controls (Supplementary Figure 10). For the most part, we found no immunopositive cells with the *T. viride* and *C. albicans* antibodies (red) (Supplementary Figure 10). The five positive samples for the *Clostridium* antibody were C1, C4, C5, C6 and C7 (amplified C1, C4, C5 and C7). Control C7 was positive for the *T. viride* antibody, but only one structure was found in this section. The peptidoglycan antibody revealed a few structures in controls C2, C4, C6, C7 and C8 (amplified C2, C6, C7 and C8). C7 was also positive with the *C. albicans* antibody. Altogether, these observations indicate that some of these commercial antibodies raised against bacteria can reveal prokaryotic-like cells and also may cross-react with structures that resemble fungi. Moreover, the burden of these structures in controls is much lower than in AD patients.

Finally, PCR analysis was carried out with DNA extracted from the seven control ERH samples. The universal primers to amplify a region of the 16 S rRNA gene of bacteria generated a robust 400 bp fragment in all seven controls tested (Supplementary Figure 11A). Indeed, the presence of bacteria was confirmed after sequencing of this fragment (Supplementary Table III). Thus, in all controls, except in C7, *Burkholderia spp* was detected, while *Clostridium spp* was found in C7. This finding was not unexpected because bacterial DNA is found in brains from control subjects^52^. Using primers designed to amplify Firmicutes DNA, one fragment of about 400 bp was observed in C8 (Supplementary Figure 11B), although the low amount of the product ruled out a sequencing analysis. A direct PCR assay similar to that described in Fig. 6D was done using primers for *Clostridium spp*. In this case, no DNA fragments were found in the seven control samples (Supplementary Figure 11C). Following an assay to detect *Bacillus* DNA, we found six out of seven control subjects with a fragment of about 1100 bp (Supplementary Figure 11D). Only in one case (C7) this DNA fragment was successfully sequenced, revealing *Streptococcus spp* (97%). Finally, no protozoal DNA was detected in the seven control ERH samples after using primers to amplify *T. gondii* SAG2 using nested PCR (Supplementary Figure 11E). All the results obtained both by immunohistochemistry and by PCR are summarized in Supplementary Table IV.

## Discussion

The suspicion that the etiology of AD is microbial in origin has been advanced by some groups^7, 9^. The main challenge with these types of studies is to first provide compelling evidence for the existence of infection in brain tissue of AD patients and then to prove that, when detected, the infection contributes to the etiology of AD, rather than a risk factor or a consequence of neurodegeneration. Regarding the first point, we have extensively analyzed potential microbial infection in AD. Accordingly, fungal macromolecules including polysaccharides, proteins and DNA can be found in peripheral blood, cerebrospinal fluid and brain tissue of AD patients^53, 54^, indicative of disseminated fungal infection. Moreover, fungal yeast-like cells and hyphae can be visualized by immunohistochemistry using specific antibodies^45, 46^. Several fungal species in brain tissue of AD patients have been identified^9, 46^. Although these species vary between patients, those belonging to the genera *Alternaria*, *Botrytis,* *Candida*, *Cladosporium*, *Cryptococcus*, *Fusarium*, *Malasezzia* and *Penicillium* are particularly prevalent^9^.

The present study shows that an evaluation of the immunoreactivity of antibodies employed to assess microbial infection is important. Some commercially available rabbit polyclonal bacterial antibodies crossreact with fungi and vice versa, and thus it is important to use an array of different antibodies. Because of this, we have tested in previous studies a variety of polyclonal antibodies raised against different fungal species, including *C. albicans*, *C. glabrata*, *C. famata*, *C. parapsilosis*, *Phoma betae*, *Penicillium notatum*, *Syncephalastrum racemosum* and *T. viride* ^45, 46, 51^. We have also used rabbit polyclonal antibodies against specific fungal components, such as chitin, enolase and β-tubulin, to detect fungal structures in AD brain sections^47^. In our opinion, the use of a single antibody against a given bacterium may lead to misleading observations.

In the present study we looked for evidence of HSV-1 reactivation in brain tissue from AD patients. Our results indicate that this reactivation does not occur in the sections examined as no expression of ICP0 or ICP5 could be found in neurons or glial cells. However, we cannot disprove that reactivation of HSV-1 is taking place in other brain regions or in other sections of ERH. Our finding that HSV-1 DNA was amplified from only one AD patient agrees with recent results^55^, however, we have only examined a small portion of the brain and only in ten patients. Previous studies examining different brain regions in many AD patients found that the proportion of positive HSV-1 DNA was 100% in some instances^12, 13^. Notably, HSV-1 and some bacteria induce the synthesis of β-amyloid and the processed Aβ peptide in culture neuronal cells and in mouse brains. This is consistent with the idea that Aβ is part of the innate immune system against microbial infections that occur in AD brains^10, 11^. In this regard, spirochetes or *C. pneumoniae* have been considered as a possible cause of the AD^7, 30, 56, 57^.

Interestingly, a variety of bacteria have been identified in human tissue of several chronic systemic pathologies, including arthritis, atherosclerosis, biliary cirrhosis and aortic aneurysms^58–62^. To our knowledge, fungal infection has not been tested in these setting. Several bacterial species have also been found in normal brain^52^. In principle, it should be possible that the colonization of a given tissue, such as CNS, by fungi could facilitate other microbial infections. Along this line, we explored the possibility that other bacterial infections are present in AD brains. Analysis to identify bacterial DNA by PCR in AD brain tissue revealed infection by several bacterial species in all ten AD patients. One of the most prominent bacteria found was *Burkholderia spp*. However, we have been unable to demonstrate the presence of spirochetes or *C. pneumoniae* by immunohistochemistry and by PCR.

The molecular basis for this interkingdom communication that can exacerbate human disease and lead to higher mortality represents an emerging field and is the subject of intense research^53, 59^. Thus, over 20% of all candidemia cases also have bacterial co-infection, which worsens the prognosis of these patients^63^. In some instances, fungal and bacterial infections are synergistic, whereas in other cases they can be antagonistic. It is quite possible that the microbial metabolites excreted by one species are beneficial or detrimental for the other species^64^. This picture is even more complicated if we consider the host immune response^65, 66^. Correspondingly, some species or their metabolites can interfere with an adequate immune response, facilitating colonization by other species. In other instances, one infection can stimulate the immune system leading to a bystander effect that may block or decrease other potential infections. Our findings clearly point to the possibility that bacterial infection can coexist with fungi in AD brains. Therefore, the picture that emerges at present is that polymicrobial infections can be detected in AD brains. These results may be important to help ascertain the precise etiology of AD and may be crucial to develop an adequate therapy for these patients.

## Materials and Methods

### Description of patients and control subjects

We analyzed frozen and paraffin-fixed samples of tissue obtained from brain donors diagnosed with AD and control individuals. All cases were diagnosed according to current neuropathological consensus guidelines^67^. Details about the age and gender of each patient are listed in Supplementary Table I. All samples used were supplied by Banco de Tejidos, Fundación CIEN (Centro de Investigación de Enfermedades Neurológicas, Madrid. Spain). The study was approved by the ethics committee of Universidad Autónoma de Madrid. The transfer of samples was carried out according to national regulations concerning research on human biological samples. For all cases, written informed consent is available. All ethico-legal documents of the brain bank, including written informed consent, were approved by an ethics committee external to the bank. The donors were anonymous to the investigators who participated in the study. Brain samples were processed as described in detail before^51^.

### Antibodies employed in this work

The following antibodies were purchased: mouse monoclonal antibody against HSV-1 ICP0, used at 1:50 dilution; mouse monoclonal antibody against HSV-1/2 ICP5 major capsid protein, used at 1:50 dilution (both from Santa Cruz Biotechnology, Santa Cruz, CA); rabbit polyclonal antibody against *Borrelia burgdorferi* (Genetex, Irvine, CA), used at 1:50 dilution; mouse monoclonal antibody against *Borrelia burgdorferi* (Abcam, Cambridge, UK), used at 1:10 dilution; rabbit polyclonal antibody against *C. pneumoniae*, which immunoreacts with the major outer porin (Biorbyt, Cambridge, UK), used at 1:20 dilution; mouse monoclonal antibody against *Chlamydia* (Abcam), used at 1:10 dilution; rabbit polyclonal antibody against *Clostridium perfringens* type D (Bioss Antibodies, Woburn, MA), used at 1:20 dilution; mouse monoclonal antibody against peptidoglycan (Thermo Fisher Scientific, Waltham, MA), used at 1:20 dilution.

Antibodies against *C. albicans* and *Trichoderma viride* were produced in our laboratory as described elsewhere^46^. Additionally, the rabbit polyclonal antibody anti-eIF4GI was produced in our laboratory as described^68^.

### Immunohistochemistry

Tissue sections from the CNS (5 μm) were fixed in 10% buffered formalin for 24 h and embedded in paraffin following standard protocols. For immunohistochemical analysis, paraffin was removed and tissues were rehydrated and boiled for 2 min in citrate buffer and then incubated for 10 min with 50 mM ammonium chloride. Subsequently, tissue sections were incubated for 10 min with 0.1% Triton X-100 in PBS and for 20 min with 2% BSA in PBS. Sections were incubated overnight at 4 °C with a primary antibody in PBS/BSA. Thereafter, sections were washed with PBS and further incubated for 1 h at 37 °C with the corresponding secondary antibody conjugated to Alexa 488 (Invitrogen, Carlsbad, CA). Sections were then incubated for 1 h at 37 °C with a third antibody in PBS, washed with PBS, and further incubated for 1 h at 37 °C with the corresponding secondary antibody conjugated to Alexa 555 (Invitrogen). Subsequently, tissue sections were stained with DAPI (Merck Millipore, Darmstadt, Germany) and samples were treated with autofluorescence eliminator reagent (Merck Millipore).

We also used the following kits: Anti-*Borrelia burgdorferi sensu stricto* IIFT (IgG), anti-*Chlamydia pneumoniae* MIF (IgG) and IIFT, *Candida mosaic* 1 (Euroimmun, Lübeck, Germany). Briefly, the primary antibody was incubated for 30 min at room temperature and the secondary antibody conjugated (to Alexa 488) was incubated for 30 min.

All images were collected and analyzed with a LSM710 confocal laser scanning microscope combined with the upright microscope stand AxioImager.M2 (Zeiss, Jena, Germany) running Zeiss ZEN 2010 software. The spectral system employed was Quasar + 2 PMTs. Images were deconvoluted using Huygens software (4.2.2 p0) and visualized with ImageJ.

### DNA Extraction from frozen CNS tissue

DNA was extracted from frozen tissue using the QIAmp Genomic DNA Isolation Kit (Qiagen, Hilden, Germany) as follows: 20 µl proteinase K (>600mAU/ml) and 180 µl of buffer ATL were added to 25 mg of brain tissue, followed by pulse-vortexing for 15 s. Digestion was carried out at 56 °C for 1–3 h with agitation. Subsequently, 200 µl of buffer AL was added to each sample followed by vortexing for 15 s and incubation at 70 °C for 10 min. A 200 µl volume of ethanol was then added to each sample followed by vortexing for 15 s. The mixture was applied to the QIAamp Mini spin column and centrifuged at 8000 rpm for 1 min. Then, 500 µl buffer AW was applied to the column followed by centrifugation at 8000 rpm for 1 min. After a final wash step with 500 µl of buffer AW2 (14000 rpm for 3 min), samples were collected in 40 µl distilled water and DNA was quantified in a NanoDrop® ND-1000 UV-Vis spectrophotometer. Negative controls included three samples of tri-distilled filtered water.

### Oligonucleotide primers

We used several sets of primers to amplify genome regions of the different microorganisms listed in supplementary Table II. Primers for herpes simplex virus type I (HSV-1) have been described by Mori *et al*.^18^ and were used to amplify the *glycoprotein D* gene. The *flagellin* gene of *Borrelia* spp was amplified with the primers described by Hudman^69^. Primers used to amplify the *MOMP* gene of *C. pneumoniae* were as described by Sriram *et al*.^70^. The location of the primers used to amplify these genes is depicted in Supplementary Figure 6.

We used sets of primers located in distinctive conserved regions to amplify the 16 S rRNA gene (see Fig. 6A). Two sets of universal primers 27F-1492R and V3-V4 were used to amplify the conserved regions V1-V9 and V3-V4, respectively. Primers to amply the 16 S rRNA from several bacteria of the Firmicutes phylum *(Listeria monocytogenes, Staphylococcus epidermidis, S. aureus, Enterococcus faecalis, Lactobacillus casei, Streptococcus pyogenes, Clostridium perfringens* and *Bacillus cereus*) were designed using the Genbank database and were aligned using ClustalW. Primers to amplify the 16 S rRNA from different *Clostridium* species (*C. perfringens, C. tetani, C. septicum, C. sordellii, C. baratii, C. novyi, C. difficile* and *C. botulinum*) were designed as above. Primers to amplify the 16 S rRNA coding region from *Bacillus* were as described by Lee *et al*.^71^. Primers used to amplify the glutamate decarboxylase (*gadB*) gene from *Escherichia coli* were as described^50^. Primers used to amplify the *SAG2* locus of *T. gondii* have also been described^40^. All oligonucleotide primers were purchased from Sigma-Aldrich (St Louis, MO).

### Nested PCR

A number of measures were used to avoid PCR contamination including the use of separate rooms and glassware supplies for PCR set-up and products, aliquoted reagents, positive-displacement pipettes, aerosol-resistant tips and multiple negative controls. DNA samples obtained from frozen brain tissues were analyzed by nested PCR using several primer pairs.

To amplify the *glycoprotein D* gene of HSV-1, the first PCR was carried out with 4 μl of DNA incubated at 95 °C for 5 min, followed by 40 cycles of 1 min at 94 °C, 1 min at 50 °C and 45 s at 72 °C. Primers used in the first PCR were forward HSV-1 external and reverse HSV-1 external. The second PCR was performed using 0.5 μl of the product obtained in the first PCR with forward and reverse HSV-1 internal primers, for 30 cycles 1 min at 94 °C, 1 min at 55 °C and 5 min at 72 °C.

The *Borrelia* spp *flagellin* gene was amplified with primers BorrFE-BorrRE and BorrFI-BorrRI. The first PCR was carried out with 4 μl of DNA incubated at 95 °C for 10 min followed by 40 cycles of 45 s at 95 °C, 30 s at 55 °C and 45 s at 72 °C. The second PCR was performed using 0.5 μl of the product obtained in the first PCR assay with forward and reverse Borr internal primers using the same conditions as the first PCR.

To amplify the *MOMP* gene, the first PCR was carried out with 4 μl of DNA incubated at 95 °C for 5 min followed by 35 cycles of 1 min at 94 °C, 1 min at 56 °C and 45 s at 72 °C. Primers used in the first PCR were forward Clam external and reverse Clam external. The second PCR was performed using 0.5 μl of the product obtained in the first PCR with forward and reverse Clam internal primers for 35 cycles of 1 min at 94 °C, 1 min at 58 °C and 5 min at 72 °C.

The human β-globin gene served as a control for DNA extraction. Primers are listed in Supplementary Table II. PCR was carried out with 4 μl of DNA incubated at 95 °C for 10 min and amplified with 42 cycles of 45 s at 94 °C, 1 min at 60 °C and 45 s at 72 °C.

Different sets of primers were used to amplify the 16 S rRNA gene (see Fig. 6A and Supplementary Table II). We first amplified the region between V1-V9. The first PCR was carried out with 2 μl of DNA incubated at 95 °C for 5 min followed by 34 cycles of 1 min at 94 °C, 1 min at 51 °C and 3 min at 72 °C. Primers used in the first PCR were 27F-1492R. The second PCR was carried out using one of the following primer sets: V3-V4, *Firmicutes*, *Clostridium* and *Bacillus*. The second PCR was performed using 2 μl of the product obtained in the first PCR with forward V3 and reverse V4 internal primers for 35 cycles 1 min at 94 °C, 1 min at 55 °C and 3 min at 72 °C. Alternatively, the second PCR was performed as above using forward Firm and reverse Firm internal primers for 40 cycles 1 min at 94 °C, 1 min at 55 °C and 3 min at 72 °C. In another reaction, forward and reverse Clost primers were used using the conditions for Firm internal primers. Also, a second PCR was performed using 2 μl of the product obtained in the first PCR with forward pB and reverse 1492 internal primers as before. We performed a direct PCR to amplify the *gadB* locus of *E. coli*. PCR was carried out with 2 μl of DNA incubated at 94 °C for 5 min and amplified with 45 cycles of 45 s at 94 °C, 1 min at 57 °C and 45 s at 72 °C. Finally, nested PCR was performed for amplification of the *SAG2* partial gene from *T. gondii* using 2 μl of DNA incubated at 94 °C for 5 min followed by 40 cycles of 45 s at 94 °C, 1 min at 59 °C and 45 s at 72 °C. The second PCR was identical to the first PCR except that the annealing temperature was 56 °C. Primers used in the first and second PCRs were forward Toxo and reverse Toxo external and forward Toxo and reverse Toxo internal, respectively. The theoretical size of the different amplicons and the primers used are listed in Supplementary Table II. Amplified DNA products were analyzed by agarose gel electrophoresis and stained with ethidium bromide. Some PCR products were sequenced by Macrogen (South Korea). The sequences obtained have been submitted to European Nucleotide Archive (ENA) with the following access number LT837521-LT837524 and LT835124-LT835152.

## Electronic supplementary material

Supplementary Information
