# Supplementary material for: Polymicrobial Infections In Brain Tissue From Alzheimer’s Disease Patients
Source: Sci Rep. 2017 Jul 17;7:5559. doi: 10.1038/s41598-017-05903-y (PMC5514053; doi:10.1038/s41598-017-05903-y)
Supplement: Supplementary file 1 — Supplementary Information [file 41598_2017_5903_MOESM1_ESM.pdf]

POLYMICROBIAL INFECTIONS IN BRAIN TISSUE FROM ALZHEIMER'S DISEASE PATIENTS

Diana Pisa[1+], Ruth Alonso[1+], Ana M. Fernández-Fernández[1], Alberto Rábano[2] and Luis Carrasco[1*]

[1]Centro de Biología Molecular "Severo Ochoa" (CSIC-UAM). c/Nicolás Cabrera, 1. Universidad Autónoma de Madrid. Cantoblanco. 28049 Madrid. Spain.

[2]Department of Neuropathology and Tissue Bank, Unidad de Investigación Proyecto Alzheimer, Fundación CIEN, Instituto de Salud Carlos III, Madrid. Spain.

[+]DP and RA contributed equally to this work.

[*]Corresponding author

Email address: lcarrasco@cbm.csic.es, telephone number: +34 91 497 84 50

Supplementary Figure 1. Detection of HSV-1 ICP0 and ICP5 in cultured HeLa cells. HeLa cells were grown in DMEM in coverslips and mock-infected or infected with HSV-1 (5 pfu/cell). Panel A: Cells were fixed and incubated with mouse antibody (1:50) against HSV-1 ICP0 (green) and rabbit antibody (1:200) against eIF4GI (red). Panel B: Immunostaining with mouse antibody (1:50) against HSV ICP5 (green) and rabbit antibody (1:200) against eIF4GI (red). DAPI appears in blue. Scale bar as shown in the figure.

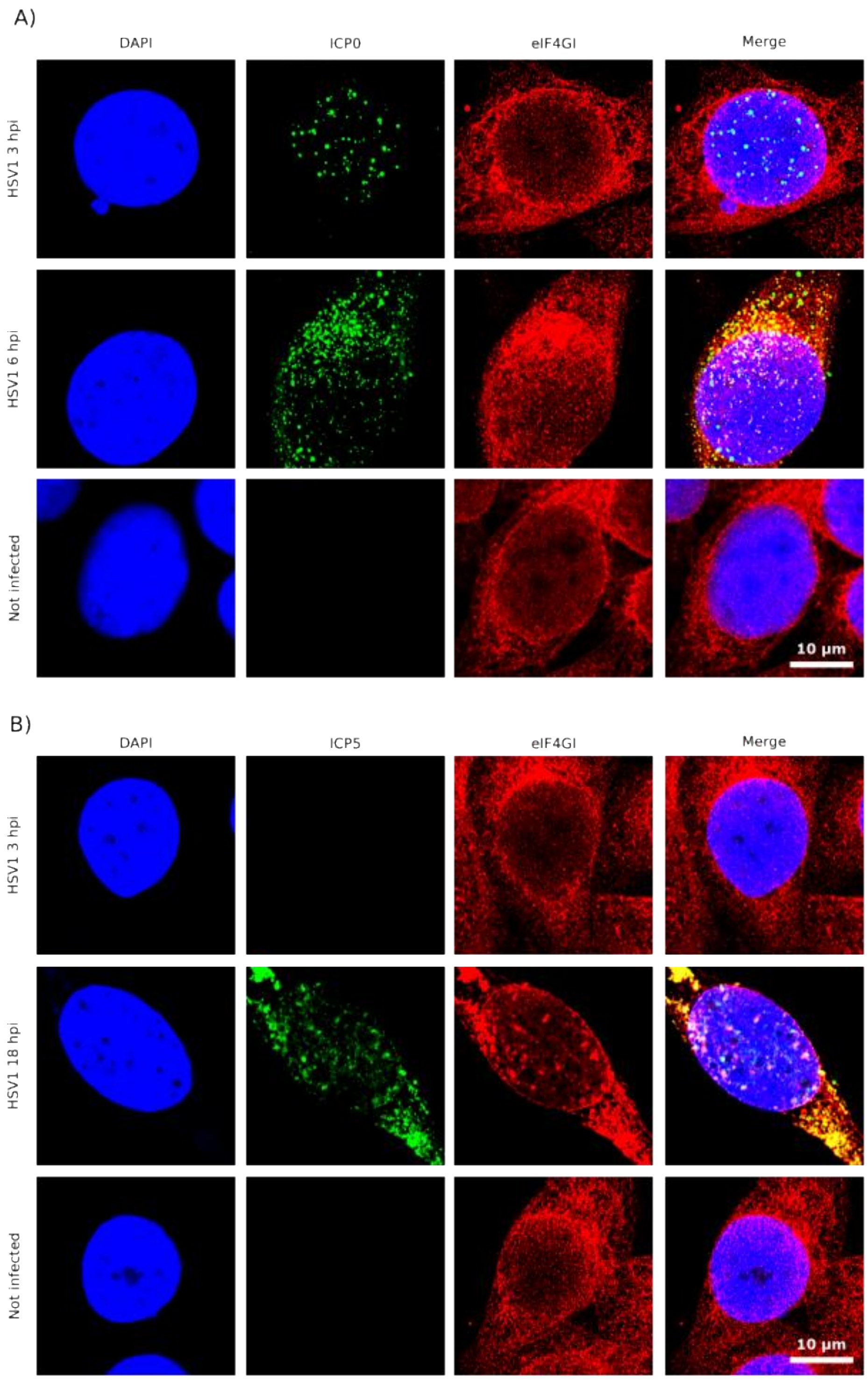
A)
DAPI
ICP0
eIF4GI
Merge
HSV1 3 hpi
HSV1 6 hpi
Not infected
10 µm
B)
DAPI
ICP5
eIF4GI
Merge
HSV1 3 hpi
HSV1 18 hpi
Not infected
10 µm

Supplementary Figure 2. Characterization of the antibodies employed in this work against *B. burgdorferi* and *C. pneumoniae*.

Panel A: The commercial Euroimmun kit (*B. burgdorferi*) was used for immunofluorescence analysis of different antibodies. Panel B: The commercial kit Euroimmun (*C. pneumoniae)* was used for immunofluorescence analysis of different antibodies. Antibody dilutions were employed as described in Materials and Methods. Scale bar as shown in the figure.

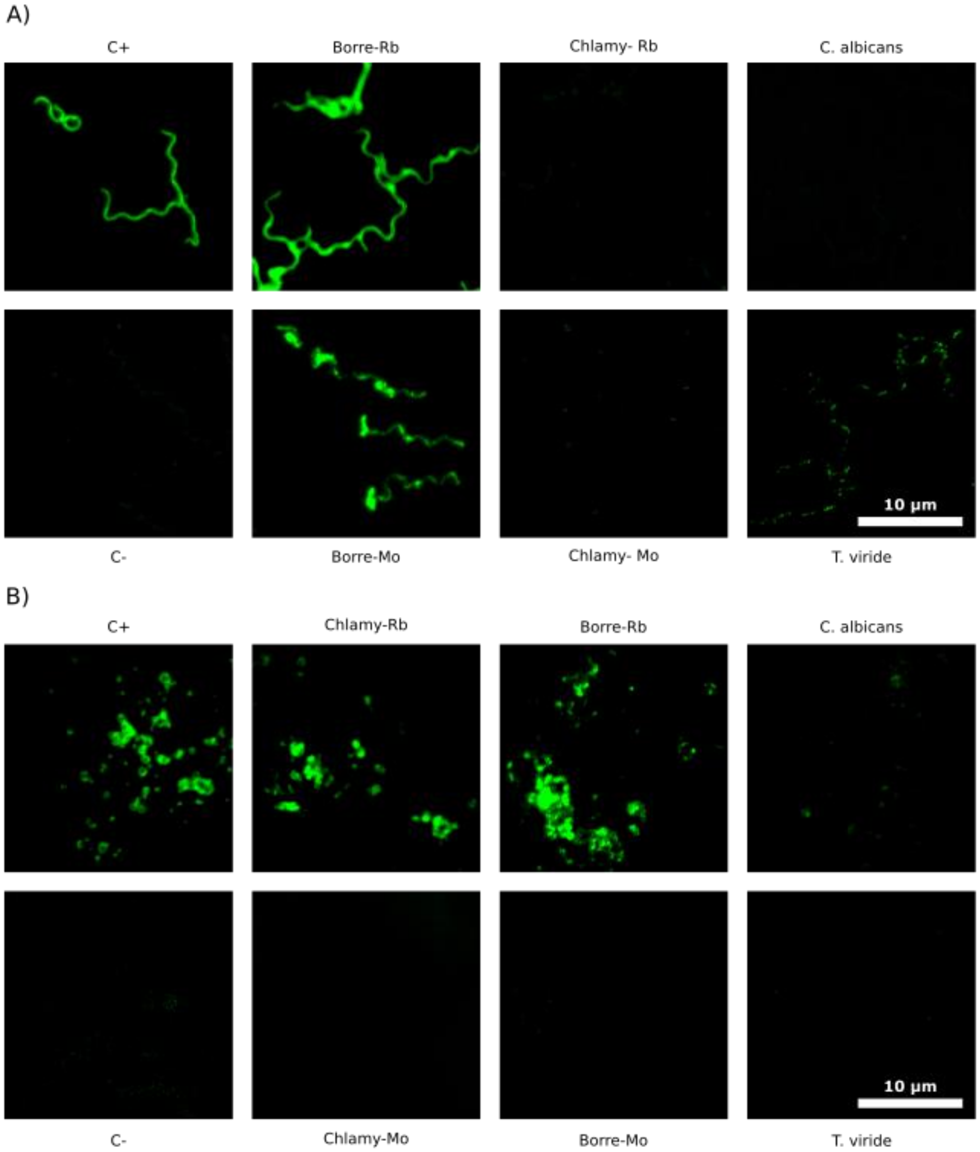

Supplementary Figure 3. Characterization of the antifungal antibodies against *Candida* employed in this work.

The commercial Euroimmun kit (*Candida*) was used for immunofluorescence analysis of two different antibodies against different *Candida* species. Antibody dilutions were employed as described in Materials and Methods. Scale bar as shown in the figure.

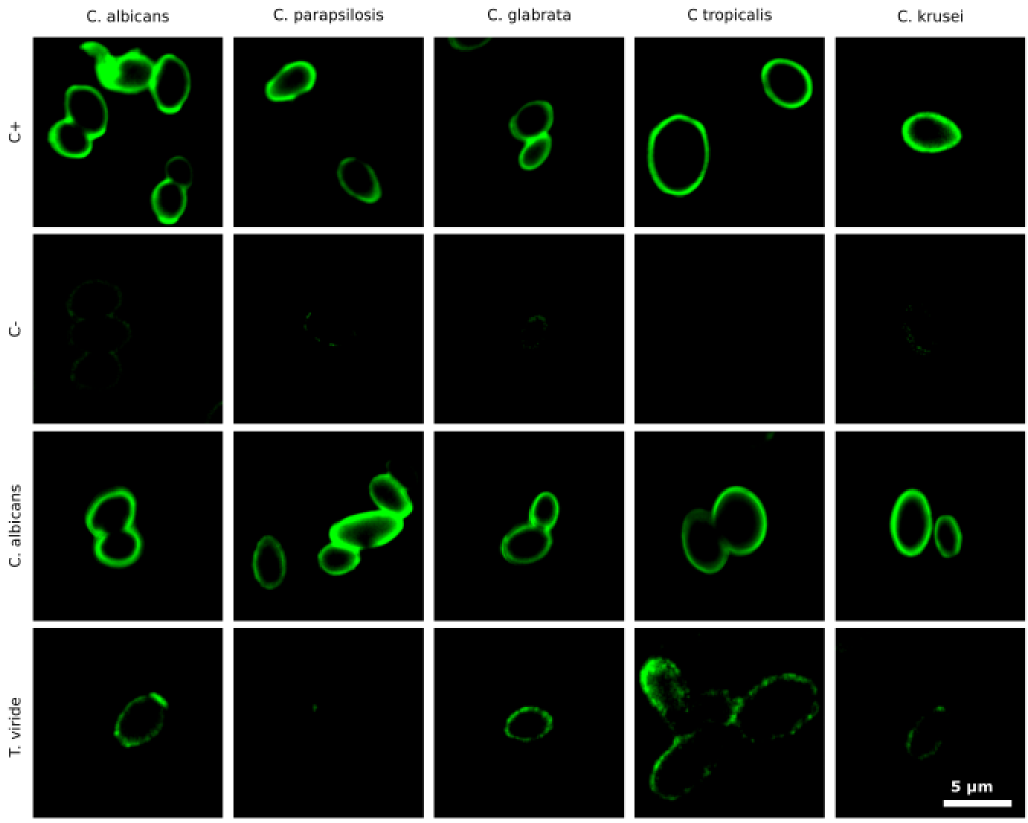

Supplementary Figure 4. Characterization of other antibodies against *Candida* employed in this work.

The commercial Euroimmun kit (*Candida*) was used for immunofluorescence analysis of different antibodies against different *Candida* species. Antibody dilutions were employed as described in Materials and Methods. Scale bar as shown in the figure.

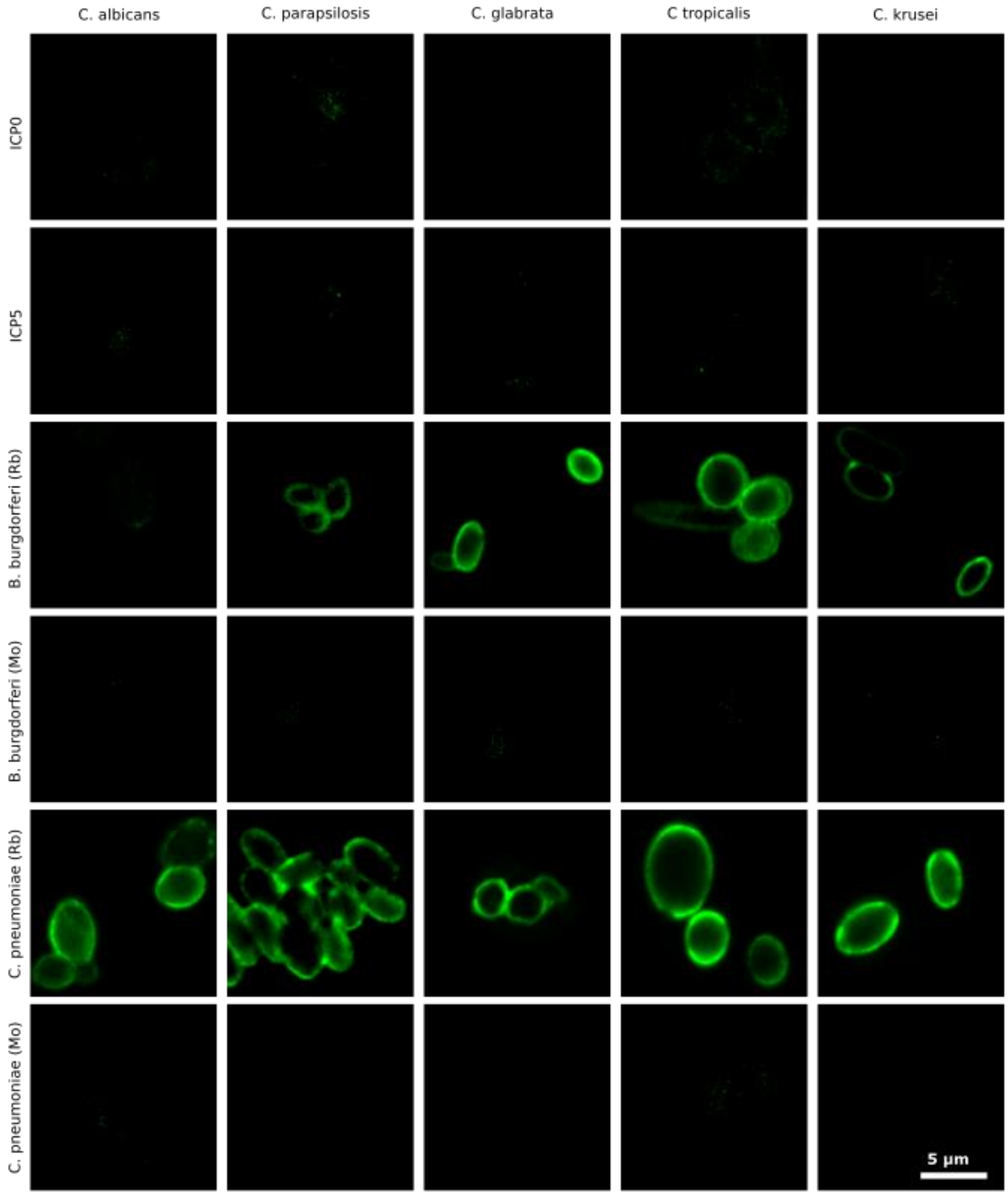

Supplementary Figure 5. Characterization of anti-*C. perfringens* and anti-peptidoglycan antibodies.

The commercial Euroimmun kit was used for immunofluorescence analysis of different antibodies against different species of *Candida* and bacteria as indicated in the Figure. Antibody dilutions were employed as described in Materials and Methods. Scale bar as shown in the figure.

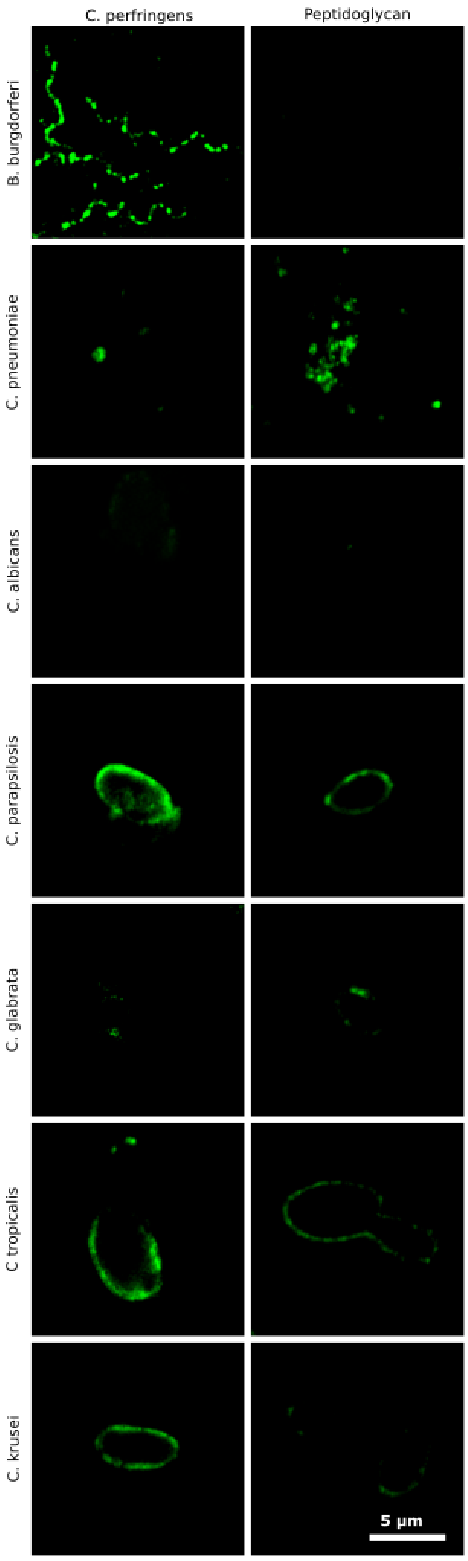

C. perfringens
Peptidoglycan
B. burgdorferi
C. pneumoniae
C. albicans
C. parapsilosis
C. glabrata
C tropicalis
C. krusei
5 µm

Supplementary Figure 6. PCR analysis of fragments amplified by each set of primers by direct or nested PCR.

Panel A: Left: Schematic representation of HSV-1 *glycoprotein D* gene and the location of the primers. Right: PCR analysis of fragment amplified with primer sets. Direct PCR assay using external Primers: HSV-1 FE-HSV-1 RE or Internal Primers: HSV-1 FI-HSV-1 RI or nested PCR combining both set of primers. DNA was extracted from HSV-1-infected HeLa cells. Panel B Left: Schematic representation of *B. burgdorferi flagellin* and the location of the primers. Right: PCR analysis of fragment amplified with primers. Direct PCR employing external Primers: Borr FE-Borr RE or Internal Primers: Borr FI-Borr RI or nested PCR combining both set of primers. DNA was extracted from *B. burgdorferi*. Panel C Left: Schematic representation of *C. pneumoniae MOMP* gene and the location of the primers. Right: PCR analysis of fragment amplified with primers. Direct PCR with external Primers: Clam FE-Clam RE or Internal Primers: Clam FI-Clam RI or nested PCR. DNA markers are indicated on the left.

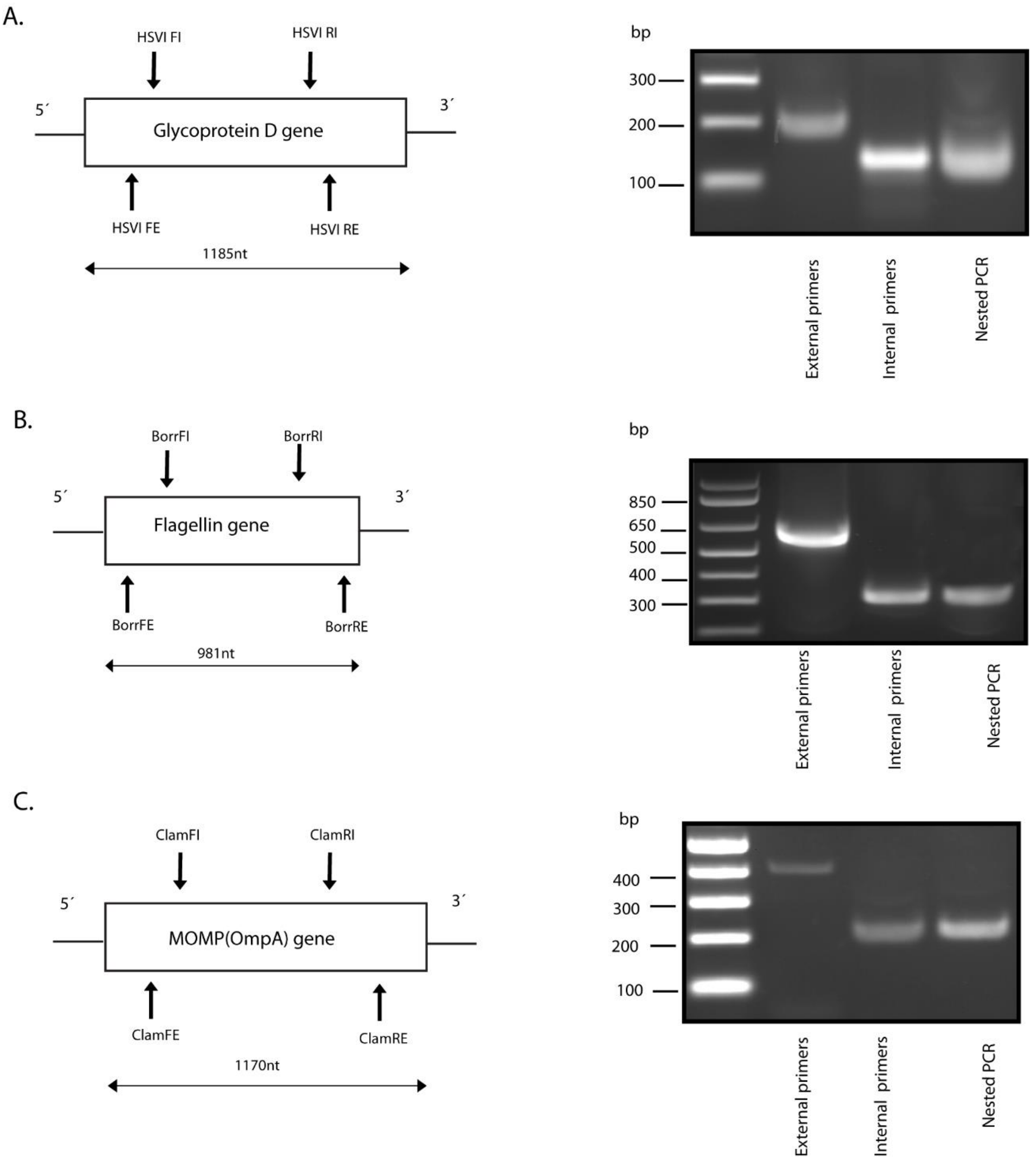

Supplementary Figure 7. HSV-1 proteins in ERH sections from eight control subjects and PCR analysis of HSV-1 DNA.

Panel A: ERH samples were incubated with mouse monoclonal antibody (1:50) against HSV-1 ICP0 (green) and rabbit polyclonal antibody (1:100 dilution) against *C. albicans* (red). The different subjects are numbered from C1 to C8 and one field is shown for each subject. In addition, four selected sections are shown at higher magnification below the eight control subjects. Panel B: samples were incubated with mouse monoclonal antibody

(1:50) against HSV-1 ICP5 (green) and rabbit polyclonal antibody (1:100) against *C. albicans* (red). DAPI staining of nuclei appears in blue. Scale bar as shown in the figure. Panel C: PCR analysis of HSV-1 and β-globin DNA in brain samples from seven control individuals. Left panel: Nested PCR analysis of DNA extracted from frozen ERH tissue using primers HSV-1 to amplify *glycoprotein D* gene. The primers employed were HSV-1 FE (forward external) and HSV-1 RE (reverse external) for the first PCR and primers HSV-1 FI (forward internal) and HSV-1 RI (reverse internal) for the second PCR. As positive control, DNA from HSV-1-infected HeLa cells was used. Right panel: PCR analysis using β-globin oligonucleotide primers. As positive control, DNA was extracted from HeLa cells. Control PCR: PCR without DNA. CE: Control of DNA extraction without DNA. DNA markers are indicated on the left.

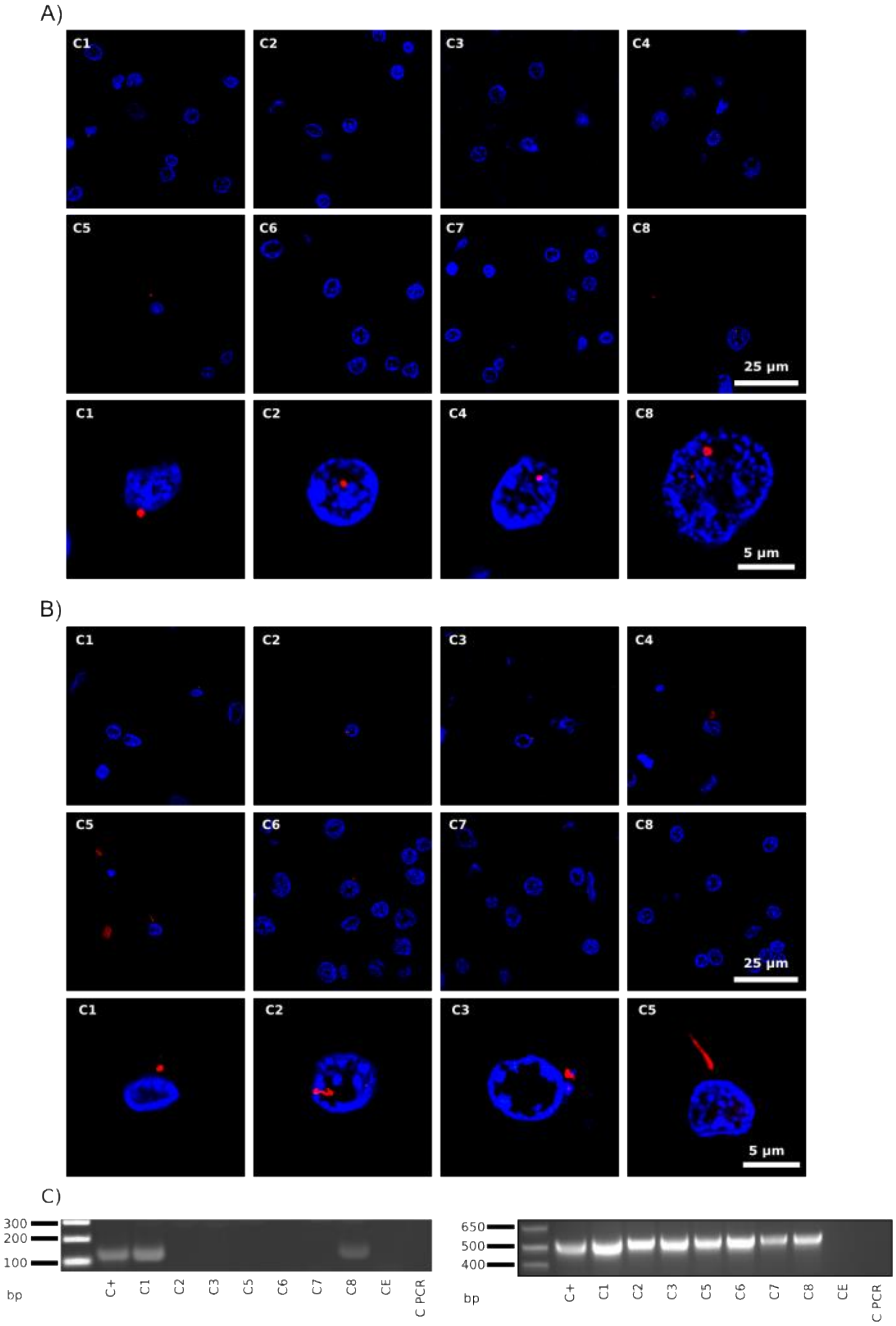
A)
C1
C2
C3
C4
C5
C6
C7
C8
25 µm
C1
C2
C4
C8
5 µm
B)
C1
C2
C3
C4
C5
C6
C7
C8
25 µm
C1
C2
C3
C5
5 µm
C)
300
200
100
bp
C+
C1
C2
C3
C5
C6
C7
C8
CE
C PCR
650
500
400
bp
C+
C1
C2
C3
C5
C6
C7
C8
CE
C PCR

Supplementary Figure 8. Detection of *Borrelia* proteins in ERH sections from eight control subjects and PCR analysis of *B. burgdorferi* DNA.

Panel A: samples were immunostained with rabbit polyclonal antibody (1:50) against *B. burgdorferi* (green) and rat polyclonal antibody (1:20) against *T. viride* (red). Panel B: samples were immunostained with mouse monoclonal antibody (1:10) against *B. burgdorferi* (green) and rabbit polyclonal antibody (1:100 dilution) against *C. albicans* (red). DAPI staining of nuclei appears in blue. Scale bar as shown in the figure. Panel C: Nested PCR analysis of *Borrelia* DNA in samples from seven control individuals. PCR analysis of DNA extracted from frozen ERH samples using primers Borr to amplify *flagellin*. The primers employed were Borr FE-BorrRE for the first PCR and primers Borr FI–Borr RI for the second PCR. As positive control, DNA from *B. burgdorferi* was used. Control PCR: PCR without DNA. CE: Control of DNA extraction without brain DNA. DNA markers are indicated on the left.

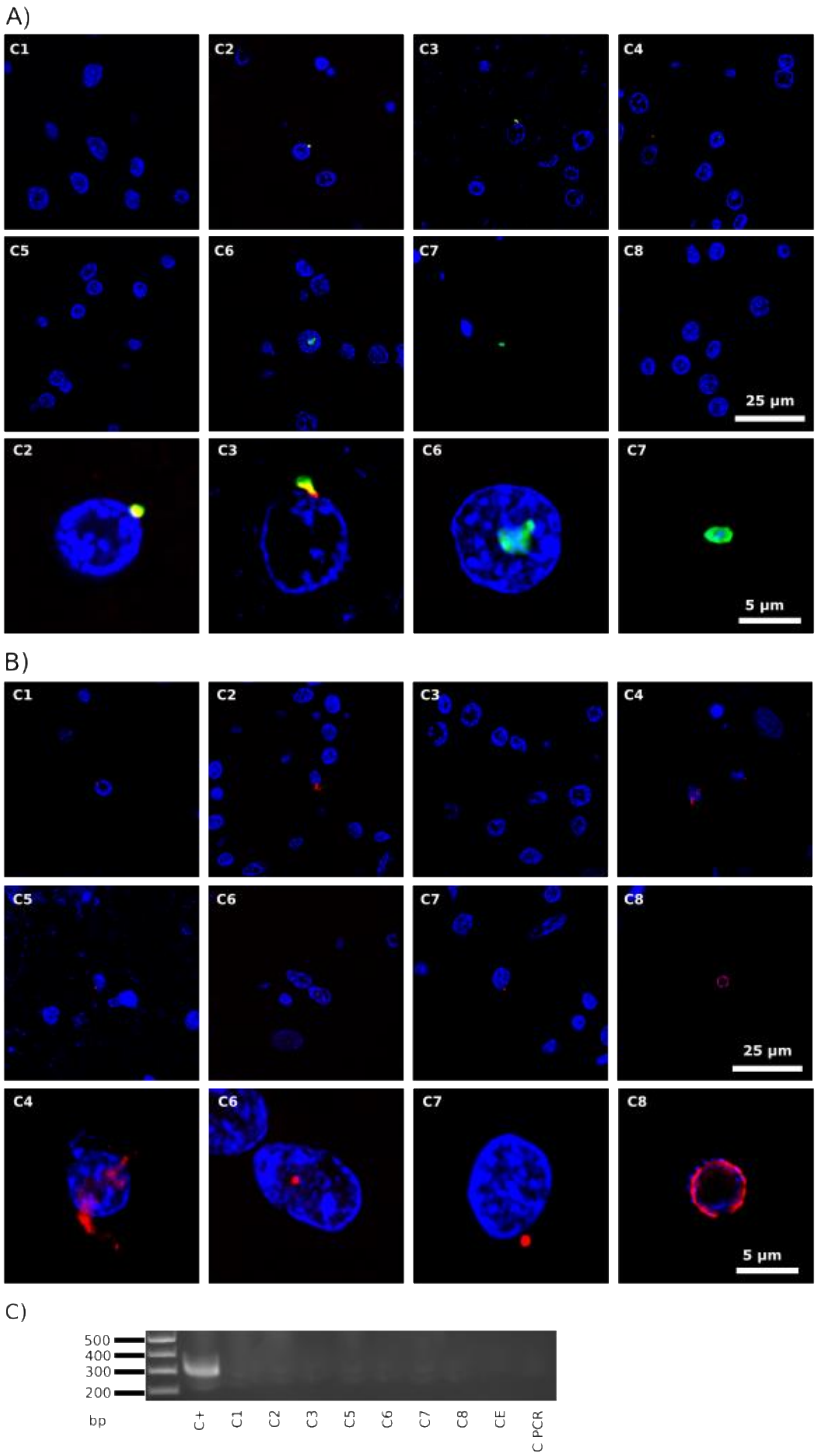
A)
C1
C2
C3
C4
C5
C6
C7
C8
25 µm
C2
C3
C6
C7
5 µm
B)
C1
C2
C3
C4
C5
C6
C7
C8
25 µm
C4
C6
C7
C8
5 µm
C)
500
400
300
200
bp
C+
C1
C2
C3
C5
C6
C7
C8
CE
C PCR

Supplementary Figure 9. Detection of *Chlamydia* proteins in ERH sections from control subjects. PCR assay of *C. pneumoniae* DNA.

Panel A: ERH sections were immunostained with rabbit polyclonal antibody (1:20) against *C. pneumoniae* (green) and rat polyclonal antibody (1:20) against *T. viride* (red). Panel B: samples were immunostained with mouse monoclonal antibody (1:10) against *C. pneumoniae* (green) and rabbit polyclonal antibody (1:100) against *C. albicans* (red) DAPI staining of nuclei appears in blue. Scale bar as shown in the figure. Panel C: Nested PCR analysis of *C. pneumoniae* DNA in brain samples from seven control individuals. PCR was carried out as described in Materials and Methods. The primers used to amplify *MOMP* were Clam FE –Clam RE for the first PCR and primers Clam FI–Clam RI for the second PCR. As positive control, DNA from *C. pneumoniae* was employed. Control PCR: PCR without DNA. CE: Control of DNA extraction without brain DNA. DNA markers are indicated on the left.

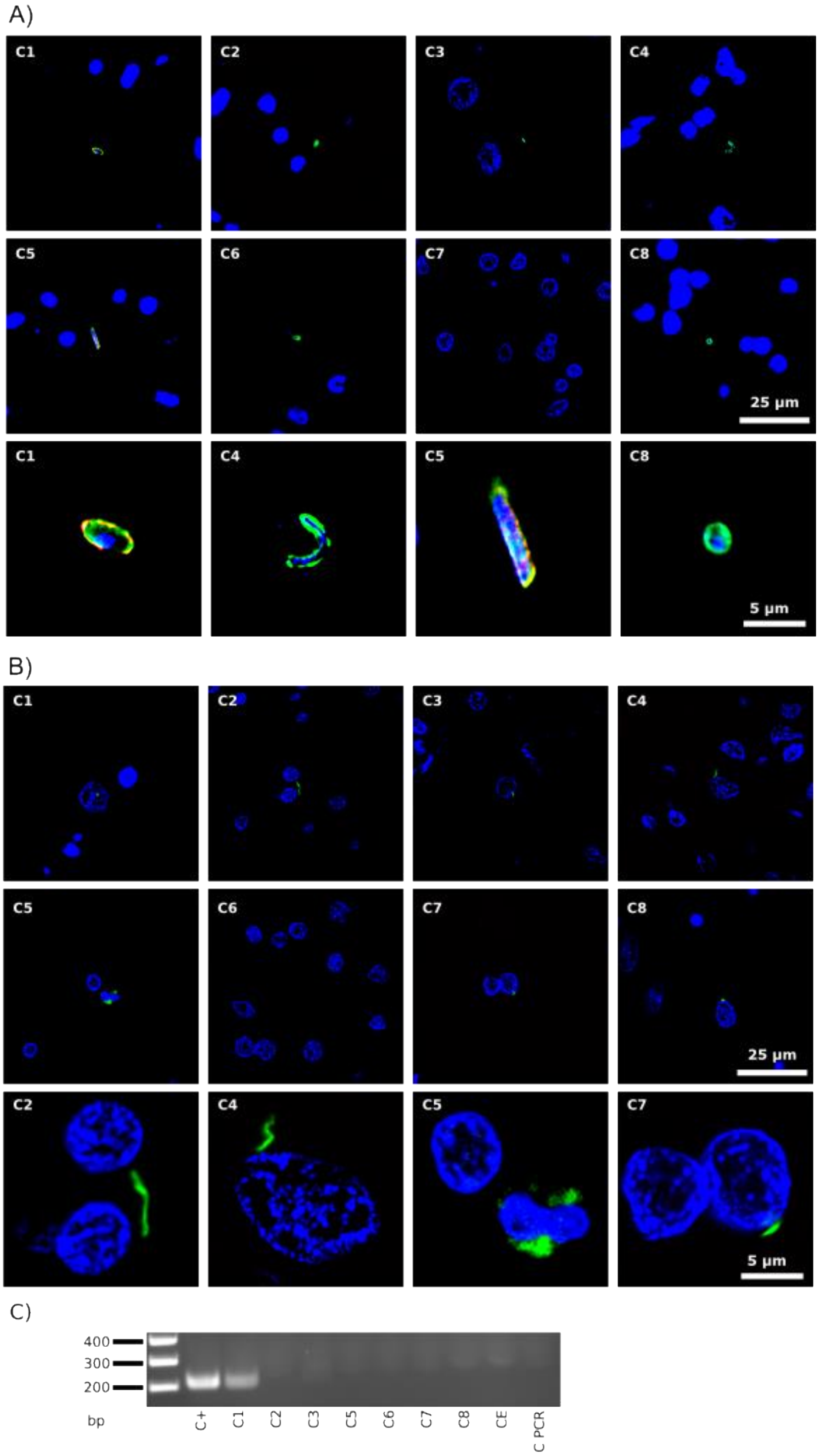
A)
C1
C2
C3
C4
C5
C6
C7
C8
25 µm
C1
C4
C5
C8
5 µm
B)
C1
C2
C3
C4
C5
C6
C7
C8
25 µm
C2
C4
C5
C7
5 µm
C)
400
300
200
bp
C+
C1
C2
C3
C5
C6
C7
C8
CE
C PCR

Supplementary Figure 10. Immunohistochemistry using anti-*Clostridium* and anti-peptidoglycan antibodies of ERH sections from control subjects.

Panel A: ERH sections were immunostained with rabbit polyclonal antibody (1:20 dilution) against *C. perfringens* (green) and rat polyclonal antibody (1:20) against *T. viride* (red). Panel B: samples were immunostained with mouse monoclonal antibody (1:20) against peptidoglycan (green) and rabbit polyclonal antibody (1:100) against *C. albicans* (red). DAPI staining of nuclei appears in blue. Scale bar as shown in the figure.

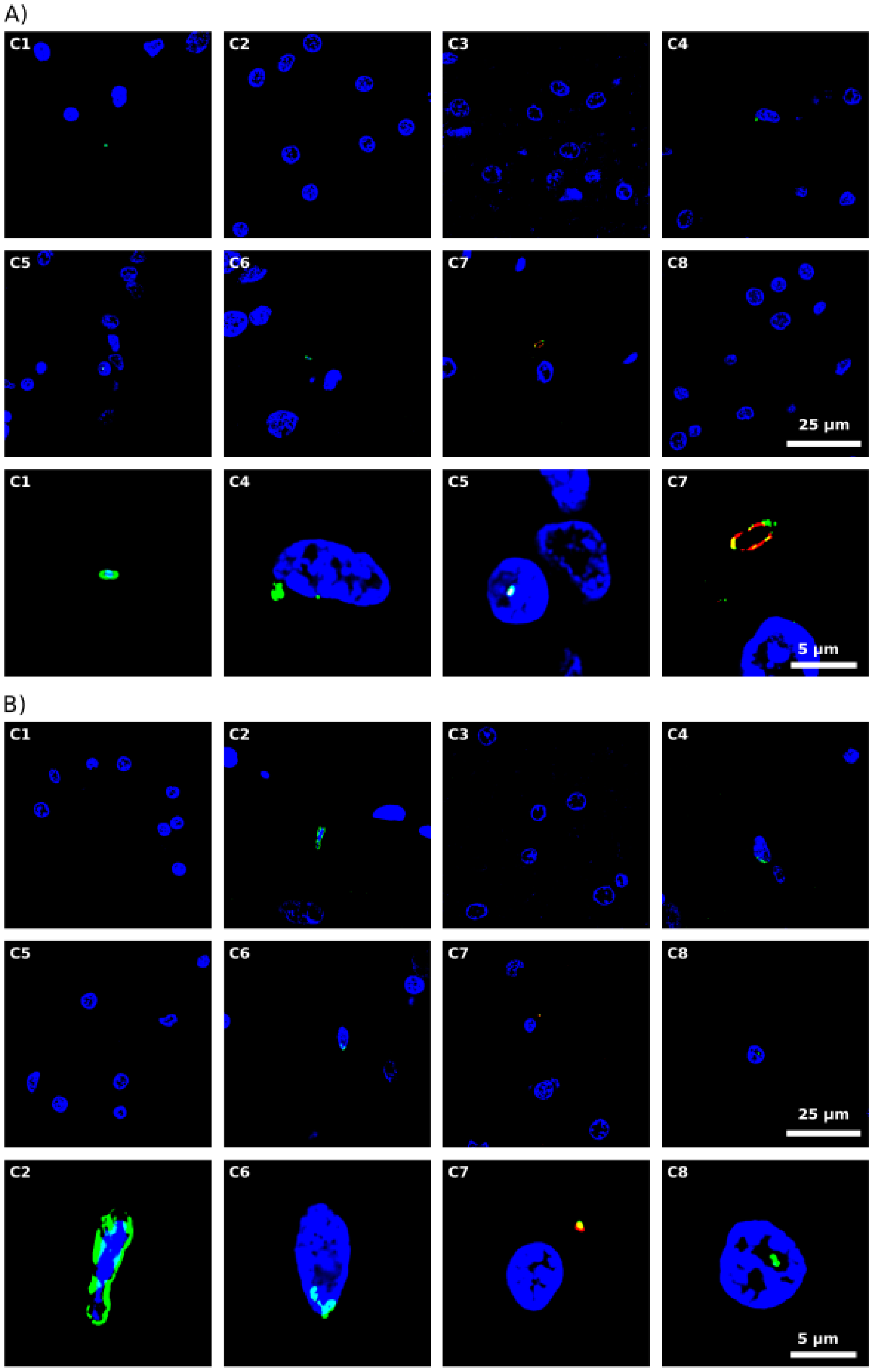
A)
C1
C2
C3
C4
C5
C6
C7
C8
25 μm
C1
C4
C5
C7
5 μm
B)
C1
C2
C3
C4
C5
C6
C7
C8
25 μm
C2
C6
C7
C8
5 μm

Supplementary Figure 11. PCR analysis of different microorganisms from seven control individuals.

Agarose gel electrophoresis of the DNA fragments amplified by PCR of DNA obtained from frozen ERH tissue from seven control subjects. Panels A–D: Nested PCR analysis of eight ERH samples to amplify 16S rRNA gene with the different sets of primers:27F-1492R (external universal primers), V3F-V4R (universal primers), FirmF-FirmR (*Firmicutes* primers), ClostF-ClostR (*Clostridium* primers), P(b) F-1492R (*Bacillus* primers). Panel A: Amplification of bacterial DNA fragment using universal oligonucleotide primers by nested PCR. The primers employed were 27F-1492R (universal primers) for the first PCR and V3F-V4R for the second PCR. All samples from control subjects show a product of about 400 bp. Panel B: Amplification of *Firmicutes* DNA fragments using specific primers by nested PCR. The primers employed were 27F-1492R for the first PCR and primers FirmF-FirmR for the second PCR. Panel C: Identification of *Clostridium* spp. DNA by Nested PCR. The primers employed were 27F-1492R for the first PCR and primers ClostF- ClostR for the second PCR. Panel D: Nested PCR assay to amplify *Bacillus* spp. DNA. The primers employed were 27F-1492R for the first PCR and P(b) F-1492R for the second PCR. Panel E: Nested PCR analysis of seven ERH samples to amplify *SAG2* partial gene (*T. gondii*). The primers used were Toxop FE and Toxop RE in the first PCR and Toxop FI amd Toxop RI for the second PCR. C -: PCR without DNA. CE: Control of DNA extraction without brain DNA. DNA markers are indicated on the left.

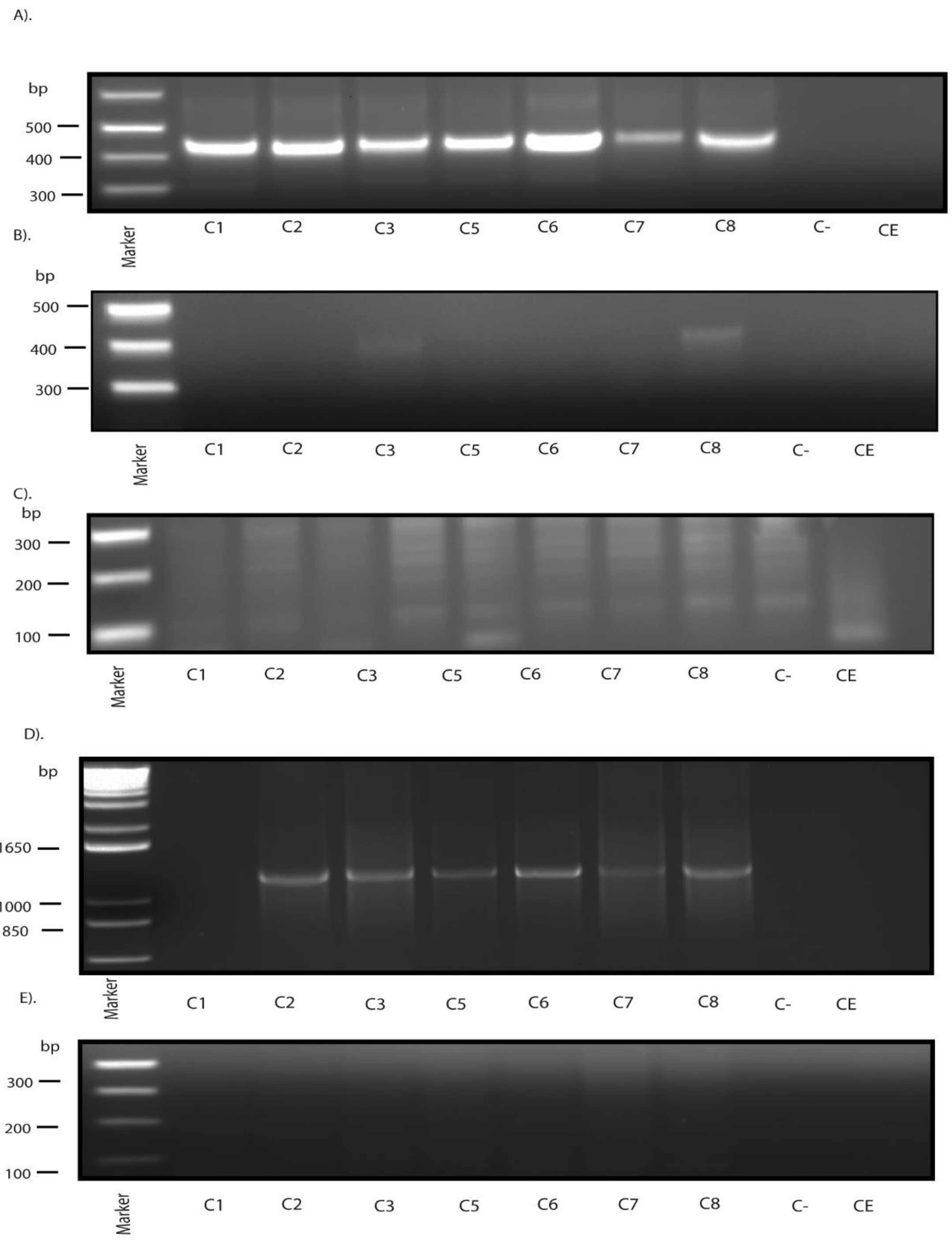
A).
bp
500
400
300
Marker
C1 C2 C3 C5 C6 C7 C8 C- CE
B).
bp
500
400
300
Marker
C1 C2 C3 C5 C6 C7 C8 C- CE
C).
bp
300
200
100
Marker
C1 C2 C3 C5 C6 C7 C8 C- CE
D).
bp
1650
1000
850
Marker
C1 C2 C3 C5 C6 C7 C8 C- CE
E).
bp
300
200
100
Marker
C1 C2 C3 C5 C6 C7 C8 C- CE

Supplementary Table I. Age and gender of AD patients and control subjects.

SAMPLE	AGE	GENDER	BRAAK TAU	SYSTEMIC DISEASES
AD1	80	FEMALE	5	
AD2	84	FEMALE	5	Arterial hypertension (AHT) Hypercholesterolemia
AD3	79	FEMALE	6	
AD4	81	FEMALE	6	AHT, Lung cancer, Diverticulosis
AD5	87	MALE	5	AHT, Dyslipemia, Deep venous thrombosis
AD6	92	MALE	6	
AD7	81	MALE	5	Dyslipemia, Diabetes Mellitus
AD8	87	FEMALE	6	
AD9	86	FEMALE	5	
AD10	62	MALE	5	Dyslipemia with poor adhesion to treatment
C1	56	MALE	1	
C2	48	FEMALE	1	
C3	63	MALE	1	No personal history of interest. Adenocarcinoma of the pancreas.
C4	78	MALE	1	
C5	55	FEMALE	0	AHT, Psoriasis in treatment.
C6	62	FEMALE	0	
C7	84	MALE	1	AHT, Restrictive cardiomyopathy, Chronic ischemic heart disease with severely revascularized 3-vessel disease.
C8	37	FEMALE	0	

Supplementary Table II. Summary of oligonucleotide primers used in this work.

Primers	sequence 5´-3´	size(bp)	gene
**HSVI FE**<br>**HSVI RE**	CATACCGGAACGCACCACACAA<br>ATCGCGGTAGCCCGGCCGTGTGTGAC	200	Glycoprotein D
**HSVI FI**<br>**HSVI RI**	CCATACCGACACACCGACGA<br>GGTAGTTGGTCGTTCGCGCTGAA	139	
**Clam FE**<br>**Clam RE**	ACAGCGTTCAATCTCGTTGG<br>GTTGCTCGAGACCATTGTACTC	410	MOMP(ompA)
**Clam FI**<br>**Clam RI**	ACACCTCTTTCTCTTGGAGCGT<br>TTGATGGTCGCAGACTTTGTT	238	
**Borr FE**<br>**Borr RE**	ACATATTCAGATGCAGACAGAGGT<br>GCAATCATAGCCATTGCAGATTGT	640	Flagellin
**Borr FI**<br>**Borr RI**	AACAGCTGAAGAGCTTGGAATG<br>CTTTGATCACTTATCATTCTAATAGC	330	
**B-globin F**<br>**B-globin R**	GGTTGGCCAATCTACTCCCAGG<br>GCTCACTCAGTGTGGCAAAG	500	B-globin
**27 F**<br>**1492R**	AGAGTTTGATCCTGGCTACAG<br>GGTTACCTTGTTACGACTT	1465	16S
**V3**<br>**V4**	CCTACGGGNGGCWGCAG<br>GACTACHVGGGTATCTAATCC	464	16S
**Firm F**<br>**Firm R**	GTGTAGCGGTGAAATGCG<br>ATCTCACGACACGAGCTGAC	360	16S
**Clostr F**<br>**Clostr R**	AGATACCCTGGTAGTCCACGC<br>GACGACAACCATGCACCACCTG	300	16S
**P(B) F**<br>**1492R**	CGATGCGTAGCCGACCTGAG<br>GGTTACCTTGTTACGACTT	1186	16S
**E.coli F**<br>**E.coli R**	CACGTTTTGGTGCGAAGTCT<br>TTGTGGACATTTTCGTCGTC	175	Gadb
**Toxop FE**<br>**Toxop RE**	GCTACCTCGAACAGGAACAC<br>GCATCAACAGTCTTCGTTGC		SAG2
**Toxop FI**<br>**Toxop RI**	GAAATGTTTCAGGTTGCTGC<br>GCAAGAGCGAACTTGAACAC	340	

Supplementary Table III. Bacterial species detected after PCR and DNA sequencing.

Subjects				
	Universal (V3-V4)	Firmicutes (V5-V6)	Clostridium (V6-V7)	Pb-1492 (V3-V9)
AD1	*Uncultured Burkholderia* (89%)	Negative	Not found	Negative
AD2	*Uncultured Sphingomonas* (85%)	Negative	*Uncultured Burkholderia* sp (96%)	Negative
AD3	*Uncultured Brevibacillus* sp (85%)	Negative	*Uncultured Burkholderia* sp (90%)	Negative
AD4	Not found	Negative	*Uncultured Burkholderia* sp (99%)	Negative
AD5	*Uncultured Burkholderia* sp (96%)	Negative	*Uncultured Burkholderia* (95%)	Negative
AD6	*Xanthomonadaceae bacterium* (90%)	Negative	*Uncultured Burkholderia* sp (99%)	Negative
AD7	*Xanthomonadaceae bacterium* (87%)	Negative	Not found	*Uncultured Streptococcus* (97%)
AD8	*Burkholderia cepacia* (98%)	Negative	*Uncultured Burkholderia* sp (98%)	*Uncultured Streptococcus (92%)*
AD9	*Uncultured Burkholderia* sp (93%)	*Staphylococcus epidermidis* (94%)	Not found	Negative
AD10	*Uncultured Burkholderia* sp (89%)	*Stenotrophomonas maltophilia* (99%)	*Uncultured Burkholderia* sp (99%)	*Staphylococcus epidermidis* (94%)
C1	*Uncultured Burkholderia* sp (95%)	Negative	Negative	Negative
C2	*Uncultured Burkholderia* sp (85%)	Negative	Negative	Not found
C3	*Uncultured Burkholderia* sp (95%)	Negative	Negative	Not found
C5	*Uncultured Burkholderia* sp (90%)	Negative	Negative	Not found
C6	*Uncultured Burkholderia* sp (96%)	Negative	Negative	Not found
C7	*Uncultured Clostridium* sp (91%)	Negative	Negative	*Uncultured Streptococcus* sp (99%)
C8	*Uncultured Burkholderia* sp (92%)	Not found	Negative	Not found

Supplementary Table IV: Summary of the results obtained.

	HSV1		BORRELIA		CHLAMYDOPHILA		BACTERIA		FUNGI	T. GONDII
	IHC	PCR	IHC	PCR	IHC	PCR	IHC	PCR	IHC	PCR
AD PATIENTS	-	+*	+†	-	+#	-	+	+	+	-
CONTROLS	-	+*	Low†	-	Low#	+**	Low	+	Low	-

IHC: inmunoshistochemistry assay

*: Positive in one AD patient and in two controls.

†: Positive for fungal and procariotic structures, but not borrelia.

#: Positive for fungal and procariotic structures, but not C. Pneumoniae.

**: Only in one control
